# Phototriggerable 2′,7-Caged Paclitaxel

**DOI:** 10.1371/journal.pone.0043657

**Published:** 2012-09-06

**Authors:** Radu A. Gropeanu, Hella Baumann, Sandra Ritz, Volker Mailänder, Thomas Surrey, Aránzazu del Campo

**Affiliations:** 1 Max-Planck-Institut für Polymerforschung, Mainz, Germany; 2 Cancer Research United Kingdom, London Research Institute, Lincoln's Inn Fields Laboratories, London, United Kingdom; 3 3rd Department of Medicine (Hematology, Oncology, and Pneumology), University Medical Center of Johannes Gutenberg-University Mainz, Mainz, Germany; Institut Curie, France

## Abstract

Three different variants of photoactivatable caged paclitaxel (PTX) have been synthesized and their bioactivity was characterized in *in vitro* assays and in living cells. The caged PTXs contain the photoremovable chromophore 4,5-dimethoxy-2-nitrobenzyloxycarbonyl (Nvoc) attached to position C7, C2' and to both of these positions via a carbonate bond. Single caged PTXs remained biologically active even at low dosages. Double caging was necessary in order to fully inhibit its activity and to obtain a phototriggerable PTX that can be applied successfully at commonly used concentrations. Irradiation of solutions containing the double caged PTX allowed dose-dependent delivery of functional PTX. Light-triggered stabilization of microtubule assemblies *in vitro* and *in vivo* by controlled light exposure of tubulin solutions or cell cultures containing caged PTX was demonstrated. Short light exposure under a fluorescence microscope allowed controlled delivery of free PTX during imaging.

## Introduction

Paclitaxel (PTX, Taxol®) is one of the most important antitumor drugs clinically used for cancer treatment [1]. It promotes tubulin assembly into stable, non-covalently polymerised structures that resist depolymerization by dilution, calcium ions, cold or microtubule disrupting drugs [2]–[3]. The alteration of the microtubule dynamics causes an extended mitotic arrest that eventually leads to cell death [4]. PTX also finds extensive *in vitro* applications, e.g. for stabilizing microtubules in studies with motor proteins and other microtubule associated proteins (MAPs), or for directing microtubule growth and therefore molecular transport in bionanotechnology approaches [5].

The structure of PTX (1) and the numbering are represented in Scheme S1. It is generally accepted that the C13 (2′R, 3′S)-Nbenzoyl-3′-phenylisoserine side-chain is essential for the cytotoxicity of paclitaxel [6]. There are three reactive hydroxyl groups in positions C2', C1 and C7 that can be used for derivatization. The reactivity of these positions towards esterification follows the sequence C2'>C7>C1. According to published literature on PTX derivatives, substitution at C1 does not significantly affect activity of PTX [7]. Esterification at C7 or C2' resulted in loss of microtubule assembly activity *in vitro* while cytotoxicity was maintained [7]. Many different variants of PTX have been synthesized since the 1980s, seeking for higher activity and selectivity towards tumors (see [7] for a recent review) and for improving its poor solubility (via BSA or polymer conjugates) or pharmacokinetics (reviewed in [6]) for clinical applications. Fluorescent PTX derivatives have also been reported [8]–[17] (and some became commercially available) and applied to imaging microtubule formation in *in vitro* experiments. Photosensitive derivatives of PTX have been proposed for light-controlled delivery of PTX and photodynamic therapy, but neither properties nor applications of these compounds have been reported [18]–[19].

A photoactivatable caged derivative of a bioactive molecule allows controlled and dynamic studies of its bioactivity by light exposure [20]–[21]. The “cage” is a chromophore unit which is mostly covalently bound to the active site of the molecule and inhibits its bioactivity. Upon light exposure the chromophore is cleaved from the structure and the activity of the biomolecule is restored. Using flash irradiation and focused lasers, the presence and concentration of the active biomolecule can be regulated with spatiotemporal resolution (down to submicrometer and microsecond scales). This approach has been applied to phototrigger ATP, glutamate and Ca^2+^ concentration jumps in cells [21]–[23], and to the photoactivation of surface-attached caged RGD peptide for triggering cell adhesion or detachment [24]–[25], amongst others.

**Figure 1:**
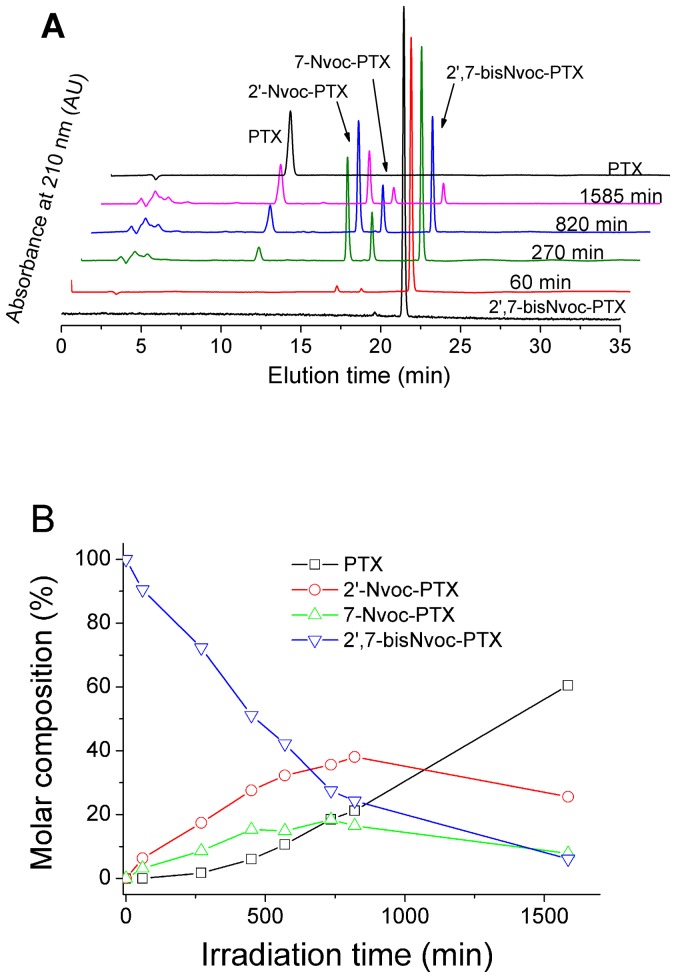
Photolytic studies. (**A**) HPLC monitoring of the photoreaction and the changes in the composition of a 1 mM solution of 2′,7-bisNvoc-PTX after irradiation at 360 nm (2.7 mW/cm^2^) with increasing dose. (**B**) Time course of the quantified molar composition of the solution, as obtained from the HPLC data.

In this work, we describe the synthesis and bioactivity of three different caged variants of PTX (2′-Nvoc-PTX, 7-Nvoc-PTX and 2′,7-bisNvoc-PTX, Fig. 1) containing the photoremovable chromophore 4,5-dimethoxy-2-nitrobenzyloxycarbonyl (Nvoc) attached to position C7, C2' and at both of these positions via a carbonate bond and we demonstrate a strategy for light-triggered stabilization of microtubule assemblies *in vitro* and in cells. Previously, a 2′ substituted photoactive caged PTX was commercially available from Molecular Probes and Invitrogen and has been used in a few cell biology studies for controlled microtubule growth [26]–[27]. However, this product was unexpectedly retracted from the market in 2003. Some of our results confirm limitations of this compound. In addition, we demonstrate substantially improved alternatives.

## Materials and Methods

### Synthesis

Paclitaxel (Xingcheng Chempharm Ltd., Taizhou, China), Nitroverantryl chloride (Nvoc-Cl, Merck, Darmstadt, Germany), dimethylamino pyridine (DMAP), methoxy-acetyl chloride and dry dichloromethane (Acros, Belgium) were purchased as p.a. quality and used without other purifications. The reactions course was followed using silicagel and RP18-silicagel TLC plates (Merck, Darmstadt, Germany).

NMR spectra were measured at room temperature with Brucker Avance 300 spectrophotometer. UV-Vis spectra were recorded with a Varian Cary 4000 UV/VIS spectrometer. PTX derivatives were purified on a JASCO HPLC 2000 series equipped with a diode array UV-Vis detection system and fraction collector, using a semi-preparative column (250×20 mm) filled with Reprosil RP18 (5 µm grain size). For the analytical measurements, a smaller column (250×5 mm) filled with the same material was used.

#### 2′-Nvoc-PTX and 2′,7-bisNvoc-PTX

A solution of 500 mg PTX (585 µmol, 1 equiv.), 242 mg Nvoc-Cl (880 µmol, 1.5 equiv.) and 107 mg DMAP (880 µmol, 1.5 equiv.) in 20 ml dry dichloromethane (DCM) was stirred overnight under inert atmosphere in dark at room temperature (RT). After 18 hrs, additional 100 ml DCM and 200 ml water were added to the reaction mixture. The organic phase was washed with water (2×100 ml), dried with magnesium sulfate and the solvent was removed in vacuum. The yellow residue was dissolved in acetonitrile containing 5% water and the products were separated by HPLC (solution A: water+0.1% TFA, solution B: acetonitrile+5% water+0.1% TFA; gradient from 50% B to 100% B in 25 min). Traces of PTX, 406 mg of 2′-Nvoc-PTX (yield 64%) and 156 mg of 2′,7-bisNvoc-PTX (yield 21%) were isolated.

#### 2′-Nvoc-PTX

^1^H-NMR (CD_2_Cl_2_, 300 MHz): δ (ppm) 1.12 (3H, s); 1.23 (3H, s); 1.77–1.80 (4H, m); 2.15–2.26 (4H, m); 2.37–2.43 (1H, m); 2.46 (3H, s); 2.50–2.58 (1H, m); 3.80 (1H, d, J = 6 Hz); 3.86 (3H, s, -O*CH_3_*); 3.91 (3H, s); 4.19 (1H, d, J = 6 Hz); 4.32 (1H, d, J = 6 Hz); 4.43 (1H, dd, J^a^ = 9 Hz, J^b^ = 6 Hz); 5.00 (1H, dd, J^a^ = 3 Hz, J^b^ = 9 Hz); 5.48 (1H, d, J = 3 Hz); 5.60 (2H, 2×d, J^gem^ = 15 Hz); 5.67 (1H, d, J = 3 Hz); 6.01 (1H, dd, J^a^ = 3 Hz, J^b^ = 9 Hz); 6.24–6.29 (2H, m); 6.96 (1H, s); 7.08 (1H, d, J = 12 Hz); 7.36–7.46 (7H, m); 7.52–7.57 (3H, m); 7.60–7.67 (1H, m); 7.71–7.74 (3H, m); 8.13–8.15 (2H, m). ^13^C-NMR (CD_2_Cl_2_, 75 MHz): δ (ppm) 10.02; 15.10; 21.20; 22.51; 23.22; 27.14; 36.05; 43.78; 46.25; 56.92; 68.03; 72.77; 72.91; 75.65; 76.15; 77.59; 79.63; 81.53; 85.06; 108.92; 110.39; 127.22; 127.68; 129.28; 129.32; 129.72; 129.88; 130.72; 132.85; 133.59; 134.27; 136.98; 143.06; 149.26; ; 154.53; 154.61; 167.42; 167.79; 168.53; 168.62; 170.56; 172.00 204.30. FD-MS: 1093.1 (M+1)^+^.

#### 2′,7-bisNvoc-PTX

^1^H-NMR (CD_2_Cl_2_, 300 MHz): δ (ppm) 1.15 (3H, s); 1.21 (3H, s); 1.80 (3H, s); 1.95–2.01 (5H, m); 2.08 (3H, s); 2.20–2.28 (1H, m); 2.39–2.47 (1H, m); 2.49 (3H, s); 2.56–2.67 (1H, m); 3.86 (3H, s, -O*CH_3_*); 3.91–3.99 (10H, m); 4.17 (1H, d, J = 6 Hz); 4.34 (1H, d, J = 9 Hz); 5.00 (1H, d, J = 9 Hz); 5.47–5.69 (7H, m); 6.02 (1H, dd, J^a^ = 3 Hz, J^b^ = 6 Hz); 6.23 (1H, t, J = 9 Hz); 6.33 (1H, s); 6.97 (1H, s); 7.01 (1H, d, J = 9 Hz); 7.09 (1H, s); 7.37–7.46 (7H, m); 7.50–7.57 (3H, m); 7.62–7.67 (1H, m); 7.70 (1H, s); 7.71–7.75 (3H, m); 8.14–8.17 (2H, m). ^13^C-NMR (CD_2_Cl_2_, 75 MHz): δ (ppm) 11.19; 14.80; 21.03; 21.71; 23.24; 26.75; 33.86; 36.01; 43.84; 47.63; 56.63; 56.90; 56.98; 67.18; 67.99; 72.78; 75.00; 75.86; 76.45; 79.26; 81.33; 84.29; 108.65; 108.90; 110.43; 111.14; 125.15; 126.43; 127.24; 127.34; 127.63; 129.11; 129.23; 129.30; 129.68; 129.83; 130.74; 132.59; 132.68; 133.33; 134.28; 137.27; 138.71; 140.14; 140.30; 141.50; 141.66; 143.49; 148.98; 149.23; 153.18; 154.31; 154.42; 154.58; 165.69; 167.30; 167.79; 168.56; 168.64; 169.62; 170.61; 204.13. FD-MS: 1332.0 (M+1)^+^.

#### 2′-MeOAc-PTX

A solution of 63 mg methoxyacetyl chloride (58 µl, 550 µmol, 1.1 equiv.) in 1 ml dry DCM was added dropwise to a solution of 426 mg PTX (500 µmol, 1 equiv.), 67 mg DMAP (600 µmol, 1.2 equiv.) in 10 ml dry DCM and the reaction mixture was stirred overnight under inert atmosphere in dark at r.t. After 18 hrs, additional 100 ml dichloromethane and 100 ml water were added to the reaction mixture and stirred for 15 min at RT. The organic phase was separated, washed with water (2×100 ml), dried with magnesium sulfate and the solvent was removed in vacuum. The white residue was dissolved in acetonitrile containing 5% water and the products were separated by HPLC (solution A: water+0.1% TFA, solution B: acetonitrile+5% water+0.1% TFA; gradient from 20% B to 100% B in 25 min). 340 mg of 2′-MeOAc-PTX (yield 67%) were isolated. ^1^H-NMR (CD_2_Cl_2_, 300 MHz): δ (ppm) 1.13 (3H, s); 1.22 (3H, s); 1.64 (3H, s); 1.75–1.85 (1H, m); 1.94 (3H, s); 1.96 (1H, s); 2.18–2.27 (7H, m); 2.37–2.54 (6H, m); 2.69 (1H, bs); 3.35 (3H, s, 2′-*CH_3_*-O-CH_2_); 3.82 (1H, d, J = 6 Hz); 4.13 (2H, dd, J = 12 Hz, 2′-CH_3_-O-*CH_2_*-); 4.17 (1H, d, J = 9 Hz), 4.29 (1H, d, J = 9 Hz), 4.98 (1H, d, J = 9 Hz); 5.60 (1H, d, J = 3 Hz); 5.67 (1H, d, J = 6 Hz); 6.03 (1H, dd, J^a^ = 3 Hz, J^b^ = 6 Hz); 6.23 (1H, t, J = 9 Hz); 6.30 (1H, s); 7.32–7.45 (8H, m); 7.49–7.57 (3H, m); 7.62–7.66 (1H, m); 7.74–7.77 (2H, m); 8.14–8.17 (2H, m). ^13^C-NMR (CD_2_Cl_2_, 75 MHz): δ (ppm) 10.02; 15.02; 21.16; 22.48; 23.16; 27.09; 36.20; 43.74; 46.28; 53.28; 58.90; 59.74; 69.91; 72.56; 72.70; 74.77; 75.66; 76.12; 76.85; 79.36; 81.52; 84.86; 127.24; 127.67; 128.98; 129.15; 129.22; 129.56; 130.03; 130.69; 132.48; 133.55; 134.12; 134.37; 137.44; 142.90; 167.22; 167.97; 168.50; 170.19; 170.52; 171.75; 204.13. FD-MS: 926.8 (M+1)^+^.

#### 2′-MeOAc-7-Nvoc-PTX, 7-Nvoc-PTX

A solution of 100 mg 2′-MeOAc-PTX (108 µmol, 1 equiv.), 44 mg Nvoc-Cl (162 µmol, 1.5 equiv.) and 20 mg DMAP (170 µmol, 1.6 equiv.) in 10 ml dry dichloromethane (DCM) was stirred overnight under inert atmosphere in dark at RT. After 18 hrs, additional 100 ml DCM and 100 ml water were added to the reaction mixture. The organic phase was separated, washed with water (2×100 ml), dried with magnesium sulfate and the solvent was removed in vacuum. The yellow residue was dissolved in acetonitrile containing 5% water and the products were separated by HPLC (solution A: water+0.1% TFA, solution B: acetonitrile+5% water+0.1% TFA; gradient from 50% B to 100% B in 25 min). 19 mg PTX, 38 mg 2′-MeOAc-PTX, 13 mg 7-Nvoc-PTX (yield 19%) and 17 mg of 2′-MeOAc-7-Nvoc-PTX (yield 23%) were isolated. The MeOAc group can be further removed by basic treatment [28]. 2′-MeOAc-7-Nvoc-PTX: ^1^H-NMR (CD_2_Cl_2_, 300 MHz): δ (ppm) 1.15 (3H, s); 1.20 (3H, s); 1.80 (3H, s); 1.91–1.2.03 (2H, m); 2.08 (3H, s); 2.18–2.27 (1H, m); 2.37–2.42 (1H, m); 2.48 (3H, s); 2.58–2.68 (1H, m); 3.37 (3H, s, 2′-*CH_3_*-O-CH_2_); 3.92 (3H, s, -O*CH_3_*); 3.95–4.01 (1H, m); 3.99 (3H, s, -O*CH_3_*); 4.06–4.20 (3H, m); 4.34 (1H, d, J = 9 Hz); 4.99 (1H, d, J = 9 Hz); 5.48–5.69 (5H, m); 5.99 (1H, dd, J^a^ = 3 Hz, J^b^ = 6 Hz); 6.21 (1H, t, J = 7.5 Hz); 6.34 (1H, s); 7.06 (1H, d, J = 9 Hz); 7.10 (1H, s); 7.32–7.47 (7H, m); 7.50–7.57 (3H, m); 7.62–7.67 (1H, m); 7.71 (1H, s); 7.73–7.76 (2H, m); 8.13–8.16 (2H, m). ^13^C-NMR (CD_2_Cl_2_, 75 MHz): δ (ppm) 11.20; 14.81; 21.03; 23.20; 26.77; 33.88; 36.07; 43.82; 46.63; 56.88; 56.99; 59.82; 67.18; 69.91; 74.60; 84.28; 108.67; 111.17; 127.26; 127.62; 129.24; 129.31; 129.62; 130.74; 137.44; 141.81; 148.99; 154.30; 154.44; 167.31; 167.57; 168.56; 169.61; 170.16; 170.60; 202.51. FD-MS: 1166.1 (M+1)^+^. 7-Nvoc-PTX: ^1^H-NMR (CD_2_Cl_2_, 300 MHz): δ (ppm) 1.15 (3H, s); 1.20 (3H, s); 1.80 (3H, s); 1.87 (3H, s); 1.93–2.01 (2H, m); 2.08 (3H, s); 2.32–2.41 (5H, m); 2.56–2.66 (1H, m); 3.65 (1H, s); 3.92–3.95 (4H, m); 3.99 (3H, s, -O*CH_3_*); 4.15 (1H, d, J = 9 Hz); 4.32 (1H, d, J = 9 Hz); 4.81 (1H, d, J = 1 Hz); 4.98 (1H, d, J = 9 Hz); 5.48–5.53 (2H, m); 5.62–5.68 (2H, m); 5.77 (1H, dd, J^a^ = 3 Hz, J^b^ = 6 Hz); 6.18 (1H, t, J = 9 Hz); 6.31 (1H, s); 7.08 (1H, d, J = 9 Hz); 7.10 (1H, s); 7.33–7.56 (10H, m); 7.62–7.67 (1H, m); 7.71 (1H, s); 7.74–7.77 (2H, m); 8.13–8.15 (2H, m). ^13^C-NMR (CD_2_Cl_2_, 75 MHz): δ (ppm) 11.17; 15.02; 21.05; 26.85; 33.25; 36.26; 43.80; 47.69; 56.76; 56.86; 56.9; 67.19; 72.83; 73.90; 79.15; 81.45; 84.23; 108.67; 111.06; 127.36; 127.59; 127.65; 129.22; 130.72; 132.45; 133.58; 134.32; 134.45; 138.92; 140.29; 141.24; 149.01; 154.35; 154.44; 167.20; 167.67; 169.66; 171.24; 173.17; 202.40. FD-MS: 1094.0 (M+1)^+^

### Photochemical studies in solution

3 ml of a 1 mM solution of caged PTX in acetonitrile containing 5% water was placed into a quartz cuvette and was exposed at 360 nm (LUMOS 43, Atlas Photonics Inc.) for different irradiation times. Aliquots of 50 µl were taken after various irradiation times, and each aliquot was split in two parts. 20 µl were diluted to 400 µl to obtain a 50 µM solution for UV-Vis characterization. The remaining 30 µl of each aliquot were injected into the HPLC (20 µl loop, analytical column in order to estimate the composition of the irradiated mixture. The concentration of each PTX derivative was determined using calibration curves (i.e. plot of concentration against HPLC signal area) recorded for PTX (210 nm), and Nvoc-PTX (380 nm).

### *In vitro* studies

#### Pre-irradiation of caged PTXs

3 ml of 100 µM caged PTX in a mixture of acetonitrile and 5 mM PIPES buffer pH 6.9 (1∶1) were irradiated for 60 min (mono-caged PTX derivatives) or for 90 min (2′,7-bisNvoc-PTX) at 360 nm, converting about 75% of the caged compounds into free PTX.

#### *In vitro* microtubule polymerization in solution

Pig brain tubulin was purified as previously described [29] and labeled with Cy5-NHS (GE Healthcare), using standard methods [30]. Labeled tubulin (9% labeling ratio) was stored in liquid nitrogen at a concentration of 230 µM. Microtubule polymerization experiments were performed in 10 µl flow chambers assembled from poly-L-lysine polyethylene glycol (PLL-PEG) treated cover slips and glass slides using double sticky tape (Tesa) [31]. The chamber was placed on an ice cold metal block and filled with 50 µl washing buffer A (80 mM PIPES, 1 mM EGTA, 1 mM MgCl_2_, 12.5 mM glycerol, 250 µg/ml BSA, pH 6.85). The chamber was then loaded with the polymerization mix, which consisted of 10 µM Cy5-labeled tubulin (9% labeling ratio) and 10 µM PTX, caged PTXs or pre-irradiated derivatives (2′-Nvoc-PTX, 7-Nvoc-PTX or 2′,7-bisNvoc-PTX) dissolved in DMSO. The final DMSO concentration in the assay buffer was 0.1% (which was also added to negative controls). The assay buffer was washing buffer A supplemented with 9 mM MgCl_2_, 1.5 mM GTP, 5 mM 2-mercaptoethanol, 50 mM KCl, 20 mM glucose, 0.7 mg/ml glucose oxidase (Serva, 22778) and 0.3 mg/ml catalase (Sigma, C40). In some experiments, the 2′,7-bisNvoc-PTX concentration was increased to 50 µM, increasing also the DMSO concentration to 0.5% (that was then also increased for controls). After loading the chamber with the polymerization mix it was sealed with VALAP (Vaseline, Lanoline, Paraffin wax, 1∶1∶1) and immediately used for imaging.

For imaging microtubule polymerization, a spinning disc confocal microscopy system (Intelligent Imaging) on a Zeiss Observer.Z1 with a 63× 1.4 NA oil immersion objective and a CCD camera (CoolSnap - Photometrix) was used. The microscope was equipped with an environment box (OKOlab) which was heated to 35°C for the *in vitro* polymerization experiments. Time lapse imaging was started immediately after placing the slide into the heated box. Images in the Cy5 channel were recorded every 10 s for 5 to 15 min with exposure times of 300 ms using 2×2 binning. For uncaging caged PTXs on the microscope, the entire field of view was illuminated for 5 s with a mercury lamp (Zeiss–HBO100) using the DAPI filter set (0.0025 µW/µm^2^ at specimen) directly after the first image of a time lapse was acquired.

To quantify the degree of microtubule polymerization, the standard deviation for all pixel intensities in a frame of a video (a measure for the contrast [32]–[33]) as calculated after background subtraction (rolling ball radius: 30 pixels, sliding paraboloid) using Image J. The time course of the standard deviation reflects the polymerization dynamics.

#### *In vitro* dynamics of individual immobilized microtubules

Individual microtubules were imaged essentially as previously described [31]. Biotinylated Cy5-labeled GMPCPPP-stabilized microtubule seeds (7% labeling ratio) were attached via neutravidin (Invitrogen) to polyethylene glycol (PEG)-biotin–coated glass coverslips being part of a flow chamber. The chamber was filled first with 50 µl washing buffer B (80 mM PIPES, 1 mM EGTA, 1 mM MgCl_2_, 0.1% methyl cellulose, 250 µg/ml BSA, pH 6.85) and then with 10 µM Cy5-labeled tubulin (7% labeling ratio) in TIRF buffer (washing buffer B supplemented with 3 mM MgCl_2_, 2 mM GTP, 5 mM 2-mercaptoethanol, 50 mM KCl, 20 mM glucose, 0.7 mg/ml glucose oxidase (Serva, 22778), 0.3 mg/ml catalase (Sigma, C40)) and 100 nM of PTX, 100 nM pre-irradiated 2′,7-bisNvoc-PTX or 50 µM 2′,7-bisNvoc-PTX dissolved in DMSO. The final DMSO concentration in the assay buffer was 0.1% (as in negative controls).

For imaging an Olympus XI71 TIRF microscope with a 100× 1.45 NA oil immersion objective and an EM-CCD camera (Cascade II - Photometrix) was used. The microscope was equipped with an environment box (EMBL Heidelberg), which was heated to 30°C. Time lapse imaging was started immediately after placing the slide into the heated box. Images in the Cy5 channel were recorded every 3 sec for 5 min with an exposure time of 200 ms using 1×1 binning.

To quantify the probability of microtubules undergoing a catastrophe (transition from growth to shrinkage), all catastrophe events leading to greater than 500 nm depolymerization in a 5 min movie were counted. The catastrophe probability was defined here as the number of all catastrophes observed in 5 min divided by the number of microtubules in the field of view. Catastrophe probabilities were determined in three independent experiments and averaged. To illustrate the time history of individual microtubules, kymographs (space-time plots) were generated for representative microtubules using the kymograph plug-in of Image J.

### Effect of caged and uncaged PTX on the microtubule cytoskeleton of living cells

#### Cell viability assays

Human cervix carcinoma cells, HeLa cells (#ACC57, DMSZ, Germany), were cultured in DMEM medium (Invitrogen) supplemented with 10% fetal calf serum (FCS, Gibco) in a humidified incubator at 37°C/5%CO_2_. The effect of PTX and derivatives on cell viability was measured by PrestoBlueTM staining (Invitrogen) according to the manufactures protocol. Briefly, HeLa cells (2×104 cells/well) were diluted in cell culture medium (DMEM, 10% FCS) and seeded in 96 well-plates (black plate, clear bottom, corning, Amsterdam, Netherlands). The culture medium was replaced after ∼16 h by compound supplemented medium (200 µl, DMEM, 10% FCS, 0.1% DMSO) or medium without compound (DMEM, 10% FCS, 0.1% DMSO) as a specific control. After 24 h and 48 h, viable cells were stained with 10 µl PrestoBlue reagent per well. Metabolically active cells reduce the cell permeable dye resazurin into fluorescent resorufin, which was measured with a fluorescence plate reader (excitation wavelength 560 nm, emission wavelength 590 nm, Tecan Infinite M1000, Austria).

#### Cell culture

HeLa cells stably expressing GFP-tubulin [34] were kept in 10 ml tissue culture dishes in culture medium (MEM, 10% FCS, 100 µg/ml penicillin/streptomycin) at 37°C, 5% CO_2_ and split 1∶10 every 3 days. For microscopy, cells were diluted 1∶10 when they reached 70% confluence, seeded (using 2 ml of cell solution) on fibronectin coated microscopy dishes (MatTek) and used for treatment with PTX and/or imaging the next day.

#### Fluorescence microscopy imaging of PTX treated cells

Cells were incubated either for 1 or 24 h in 2 ml imaging medium (culture medium with 40 mM HEPES, pH 7.15) (as control) or in 2 ml imaging medium containing 0.1% DMSO and 10 µM PTX, caged PTX or pre-irradiated caged PTX. In some experiments 0.5% DMSO and 50 µM 2′,7-bisNvoc-PTX was used. Images were recorded using the spinning disk confocal microscope described above (GFP channel, 400 ms exposure time, 2×2 binning, 37°C).

#### Photolysis of 2′,7-bisNvoc-PTX in cell culture

Cell culture medium was exchanged to imaging medium containing 0.5% DMSO or 50 µM 2′,7-bisNvoc-PTX just before each experiment. The sample was irradiated for 5 sec using a mercury lamp through a DAPI filter set (full field of view, 0.0025 µW/µm^2^ at specimen). Images of the cells were recorded shortly before and 1 h after irradiation.

#### Determination of the mitotic index of HeLa cells using FACS analysis

Cells at 60% confluence were incubated for 20 h in 2 ml cell culture medium containing 0.1% DMSO, 100 nM PTX, 100 nM pre-irradiated 2′,7-bisNvoc-PTX or 50 µM 2′,7-bisNvoc-PTX. For flow cytometry analysis for each individual assay approximately 0.5·10^6^ cells were fixed in 70% ethanol. For the stain against phospho-histone the cells were centrifuged at 2000 rpm and washed in PBS+2% FCS and resuspended in 0.1% Triton X-100 in PBS/2% FCS. The primary antibody (mouse anti-phospho histone H3 serine 10, Cell Signalling Inc, cat. no. 9701) was used in a 1∶200 dilution for 1 h at room temperature. After washing in PBS/2% FCS the cells were centrifuged and resuspended in secondary antibody (goat anti-mouse AF647 labeled antibody, Life Technologies, cat. no. A21235) used in a 1∶200 dilution for 30 min in the dark, again diluted in PBS/2% FCS and washed as before. Cells were then resuspended in 50 µl of RNasA solution (100 µg/ml) and 200 µl propidium iodide (50 µg/ml) for 30 min before acquisition on a LSR Fortessa (BD Biosciences). The phospho-histone H3 AF647 fluorescence was detected using a 670/14BP filter and excitation from a 633 nm laser and the propdium iodide fluorescence was detected using a 610/20BP filter and excitation from a 561 nm laser. Data analysis was performed using FlowJo software (TreeStar INC).

## Results and Discussion

### Synthesis

2′-Nvoc-PTX, 7-Nvoc-PTX and 2′,7-bisNvoc-PTX were prepared by reaction of PTX with Nvoc-Cl under different conditions (Scheme S2). The reaction of PTX with Nvoc-Cl in low excess afforded 2′-Nvoc-PTX as well as traces of the disubstituted 2′,7-bisNvoc-PTX. Increasing the excess of Nvoc-Cl in the reaction mixture raised the yield of 2′,7-bisNvoc-PTX up to 21%. Mass spectrometry and the appearance of the characteristic signals for the Nvoc unit in the NMR spectra confirmed the molecular structure of the substituted PTX. In addition, 2′ substitution shifted the C2' proton signal in the ^1^H-NMR spectrum from 4.8 to 5.6 ppm (see NMR spectra in Fig. S8), in agreement with other reported C2' substituted PTX derivatives [35]. For the synthesis of 7-Nvoc-PTX, the 2′-OH group was first protected as methoxy-acetate (MeOAc) ester [28] before reaction with Nvoc-Cl. A stoichiometric amount of DMAP was required for obtaining decent yields. The chemical shift of the C7 proton shifted from 3.78 to 5.5 ppm upon Nvoc functionalization (see NMR spectra in Fig. S8). Significant deprotection of the MeOAc ester occurred during the reaction, so that 7-Nvoc-PTX was directly isolated from the reaction mixture.

All caged derivatives of PTX were stable as solid and as DMSO (dry) solution and as acetonitrile (containing 5% water) solutions (10 mM). No hydrolysis was detected upon storage in 80 mM PIPES buffer (pH 6.9) and in dark over one month (Fig. S1). Despite the fact that the Nvoc substitution increased the hydrophobicity of the PTX moiety, the caged PTX derivatives have a slightly better water solubility than PTX. In fact, 10 mM DMSO solutions could be diluted with cell culture medium to 1∶1000 and the PTX derivatives remained in solution for at least 72 h.

### Photochemical properties

The photolysis of the compounds 2′-Nvoc-PTX, 7-Nvoc-PTX and 2′,7-bisNvoc-PTX was followed by UV and HPLC analysis of the correspondent solutions after exposure to light at λ_max_ = 360 nm (2.7 mW/cm^2^). UV spectra of the irradiated solutions showed hypsochromic and hypochromic shifts of the absorption maxima (Figs. S2, S3 and S4) and the appearance of a maximum with low absorbance at 396 nm. These changes agree with reported data on the photocleavage of Nvoc derivatives and formation of the nitroso product during photolysis as a result of an intramolecular redox reaction [36]–[37]. The generation of free PTX was confirmed for all three cases by HPLC (Figs. S2, S3, S4). The chemical yields for PTX release varied with the caging position: 52% for the 2′-Nvoc-PTX, 89% for the 7-substituted and 61% for the double caged PTX. The photocleavage of the double caged compound 2′,7-bisNvoc-PTX yielded a mixture of the mono-substituted species as intermediates that were further photolyzed to free PTX. HPLC analysis of 1 mM photolyzed solutions (Fig. 1A) confirmed the presence of 2′-Nvoc-PTX (retention time, r.t. = 16.7 min) and 7-Nvoc-PTX (r.t. = 18.2 min) together with the starting compound 2′,7-bisNvoc-PTX (r.t. = 21.3 min) and the fully deprotected PTX (r.t. = 11.1 min). The ratio of these compounds in the irradiated mixture changes with the exposure dose. Figure 1B (and table S1) shows the compositional evolution of the mixture with increasing irradiation time as obtained by HPLC analysis. According to these results, the photocleavage of the carbonate at position 7 was more effective than the carbonate at position 2′. This suggests that Nvoc protected cyclic hydroxyls may be cleaved more effectively than linear ones. However, the influence of other structural factors (i.e. steric ones) in the photolytic mechanism cannot be ruled out. Note that the long irradiation times are due to the low intensity of the lamp and the high concentration (1 mM) of the caged PTX solution used for the experiment (as required for later HPLC analysis and identification of photoproducts). In the light microscope 1000 times higher irradiation power densities are reached, allowing correspondingly shorter irradiation times for uncaging.

### Interaction of caged and uncaged PTX with tubulin *(in vitro)*

**Figure 2:**
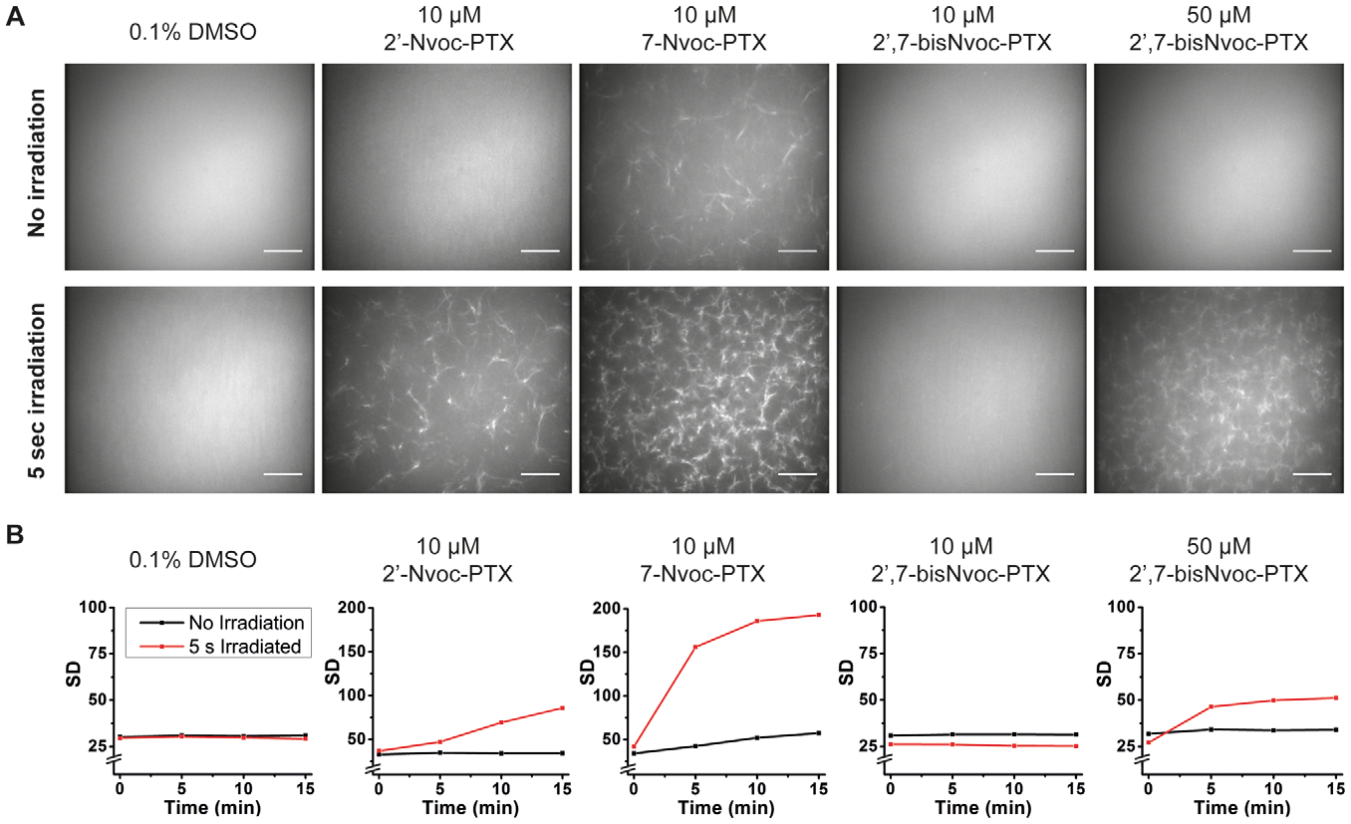
*In vitro* polymerization of microtubules. (**A**) Fluorescence microscopy images of a solution containing 10 µM Cy5-labeled tubulin and 10 or 50 µM caged PTXs, as indicated. Images were taken 10 min after triggering microtubule polymerization by a temperature increase to 35°C and either no (upper panel) or 5 sec UV irradiation (lower panel). 0.1% DMSO was added to the negative control, because stock solutions of caged PTXs were dissolved in DMSO. Scale bars: 20 µm. (**B**) Quantification of the degree of microtubule polymerization. As a measure for contrast, the standard deviation of all pixel intensities per image is shown as a function of time after the temperature increase. See Fig. S5 for *in vitro* polymerization experiments with pre-irradiated solutions of the caged PTXs.

The interaction of the caged PTXs with purified tubulin before and after irradiation was tested in a microtubule polymerization assay. A 10 µM Cy5-labeled tubulin solution containing 10–50 µM caged PTXs was exposed at 360 nm for 5 s, incubated for 10 min at 35°C, and imaged by fluorescence microscopy (Fig. 2). In controls, PTX was not added (Fig. 2A left) or irradiation was omitted (Fig. 2A top). At the chosen tubulin concentration, nucleation of microtubules was inefficient but it was strongly promoted by addition of 10 µM PTX (Fig. S5). In the presence of 10 µM 2′-Nvoc-PTX no microtubule nucleation was observed, whereas a loose meshwork of microtubules was visible after exposure (Fig. 2A bottom), indicating photogeneration of free PTX. Only a fraction of caged PTX was uncaged under our irradiation conditions as shown by comparison to the stronger nucleation effect of 10 µM PTX. This is most likely due to fast diffusion (and resulting dilution) of the locally uncaged PTX compounds. 10 µM 7-Nvoc-PTX showed a weak microtubule nucleating activity before exposure (Fig. 2A top), indicating that the Nvoc substitution at position C7 does not fully inhibit the interaction with tubulin. Irradiation significantly increased the nucleating activity of 7-Nvoc-PTX. 2′,7- bisNvoc-PTX did not lead to microtubule nucleation, even at 50 µM concentration (Fig. 2A top right). For quantifications of the nucleation effects see Figure 2B. Irradiation of tubulin solutions with 10 µM 2′,7-bisNvoc-PTX showed only a faint nucleating activity (Fig. 2A bottom, Movie S1). However, the same exposure dose in the presence of 50 µM 2′,7-bisNvoc-PTX caused effective microtubule nucleation (Fig. 2A bottom right, Movie S2). This can be understood considering that light exposure of the double caged PTX generates the monocaged as reaction intermediates. Hence, doses higher than for single caged derivatives are required, to generate the same amount of fully uncaged PTX. Pre-irradiated, uncaged PTXs derivatives strongly promoted efficient nucleation similar to PTX (Fig. S5). In summary, 2′-Nvoc-PTX and 2′,7-bisNvoc-PTX are efficiently caged variants of PTX that only cause effective microtubule nucleation *in vitro* upon light exposure.

### Effect of caged and uncaged PTX on cultured cells

**Figure 3:**
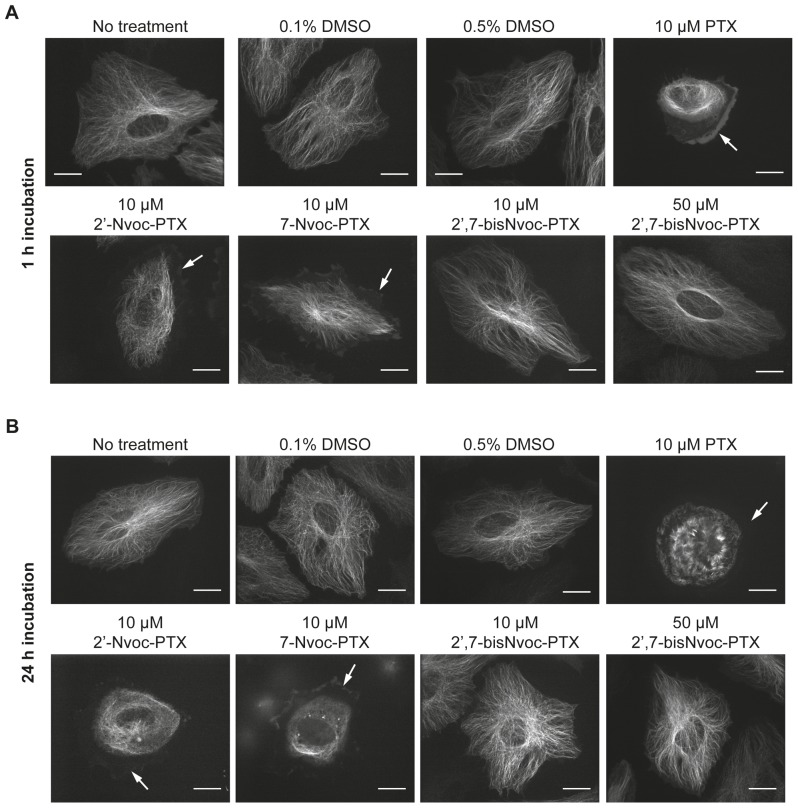
Effect of the presence of PTX and caged PTXs on the microtubule cytoskeleton of living cells. Fluorescence microscopy images of HeLa cells stably expressing GFP-tubulin imaged after 1 h (**A**) and 24 h (**B**) incubation in medium containing DMSO, PTX and caged derivatives at the indicated concentrations. White arrows indicate the cell edge deprived of microtubules. Scale bars: 20 µm. See Fig. S5 for images of assays with pre-irradiated compounds.

We then tested the effect of caged PTXs on the microtubule cytoskeleton of living cells. HeLa cells were incubated with PTX and caged variants. In the absence of PTX, cells displayed an astral array of microtubules (Fig. 3, No Treatment). When treated with PTX for 1 h, some cells detached from the substrate and those that remained adherent showed a strongly disorganized, contracted microtubule cytoskeleton with bundled microtubules. Cell edges were free from microtubules (Fig. 3A, white arrows). Adherent cells treated with 2′-and 7-Nvoc-PTX also displayed a disorganized microtubule cytoskeleton, even if not as drastic as in cells incubated with free PTX (Fig. 3A). In contrast, the microtubule cytoskeleton of cells incubated with the double caged 2′,7-bisNvoc-PTX showed a normal morphology, even at elevated concentrations (Fig. 3A bottom right) and after incubation for 24 h (Fig. 3B). These results indicate that both single caged PTXs are cytotoxic and, therefore, a single Nvoc substitution does not fully inhibit PTX activity in living cells. These results reflect an important limitation of single caged PTXs for experiments with living cells that was not detectable in the experiments with purified tubulin. This suggests that PTX might have an additional effect in cells which is not based on its microtubule stabilizing function and that single caging does not block this unknown activity, although it interferes with the microtubule stabilizing activity.

Experiments performed with pre-irradiated solutions of all caged PTXs showed a disorganized cytoskeleton characteristic for the presence of free PTX (Fig. S6). In conclusion, our experiments demonstrate that only double caging PTX at the 2′ and 7 positions effectively inhibits it activity in living cells when used at micromolar concentrations typically used for experiments with cells and that biological activity is recovered after uncaging by irradiation.

**Figure 4:**
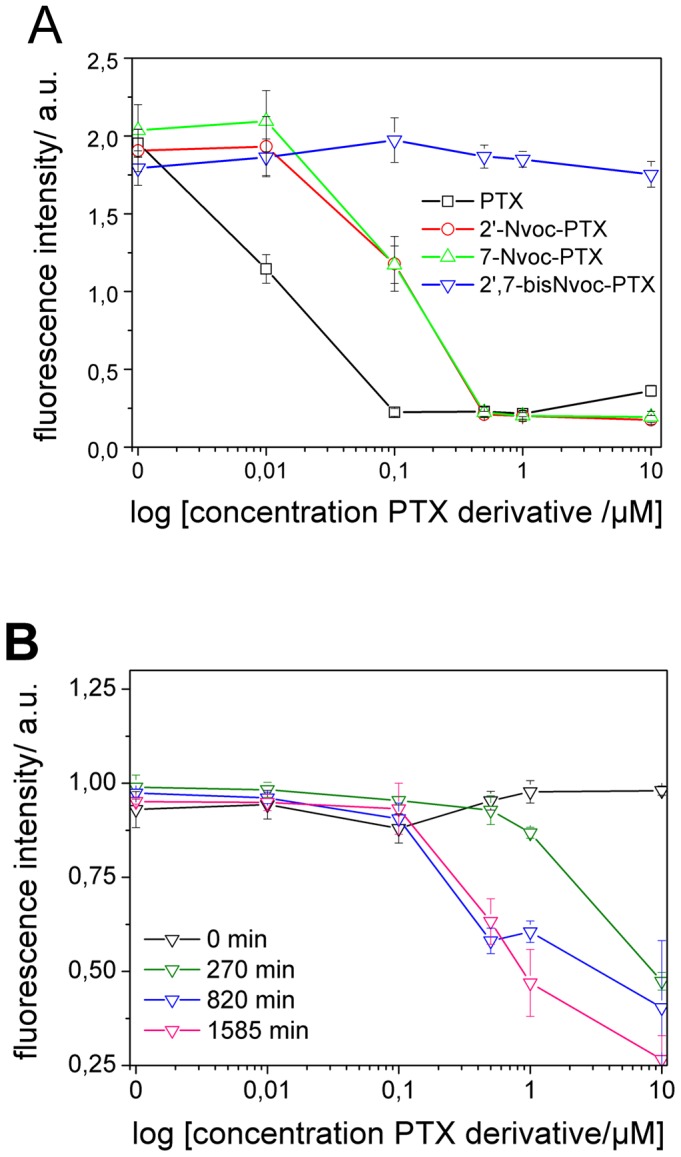
Cell viability in the presence of PTX derivatives at different concentrations. (**A**) HeLa cells were incubated with PTX and caged PTX derivatives at concentrations between 0.01 and 10 µM. After 48 hours cultivation, cells were stained with PrestoBlue™ reagent. Metabolically active cells reduce the cell permeable dye resazurin into fluorescent resorufin, providing a quantitative measure of viable cells (see “Materials and Methods” section for details). (**B**) The viability of cells incubated with pre-irradiated solutions of 2′,7-bisNvoc-PTX.

The observed cytotoxic effects of the caged PTXs from the single cell experiments were further confirmed by evaluating the viability of a liquid HeLa cell culture after 48 hours incubation with PTX and caged PTX derivatives at concentrations between 0.01 and 10 µM (Fig. 4A). PTX significantly reduced cell viability at a concentration of 0.1 µM. This value is in good agreement with reported cytotoxicity levels by other groups [38]. The double caged derivative did not show any cytotoxicity at concentrations up to 10 µM, indicating that the double caging effectively inhibited the activity of the drug. Single caged PTXs showed a ∼50% reduction in vitality at 0.1 µM, indicating a reduced but still significant cytotoxicity (a ∼50% reduction of vitality for uncaged PTX occurred at concentration 0.01 µM). This might explain why not many applications were reported for the 2′Nvox-PTX derivative that was previously commercially available. Single cell imaging and population vitality results demonstrate that only double caging of PTX suppressed cytotoxicity over the entire range of concentrations tested.

We measured also the vitality of HeLa cells treated with defined concentrations of pre-irradiated solutions of 2′,7-bisNvoc-PTX (Fig. 4B). The irradiated solutions contain a mixture of 2′,7-bisNvoc-PTX, free PTX, and the 2′- and 7-caged derivatives in different ratios (see Fig. 1B and Table S1 for the precise composition). A reduction in vitality was visible already at the shortest irradiation time, indicating light-triggered release of cytotoxic PTX and 2′-Nvoc-PTX. The concentration at which cell vitality is reduced decreased with increasing irradiation time, as expected. Vitality levels of 0.5 were reached for the longer irradiation times at a concentration of 5 µM 2′,7-bisNvoc-PTX, in accordance with the values observed for PTX solutions at the same concentration. These results confirm the presence of functional uncaged PTX in the pre-irradiated solutions (as identified by HPLC, Fig. 1A), and demonstrate light-controled delivery of PTX.

### Further characterization of 2′,7-bisNvoc-PTX

Since our previous experiments indicated that only the double caged PTX has no cytotoxic effect on cells and can be efficiently uncaged, we further tested it for potential minor effects, using a very high concentration of caged compound next to a low concentration of PTX.

**Figure 5:**
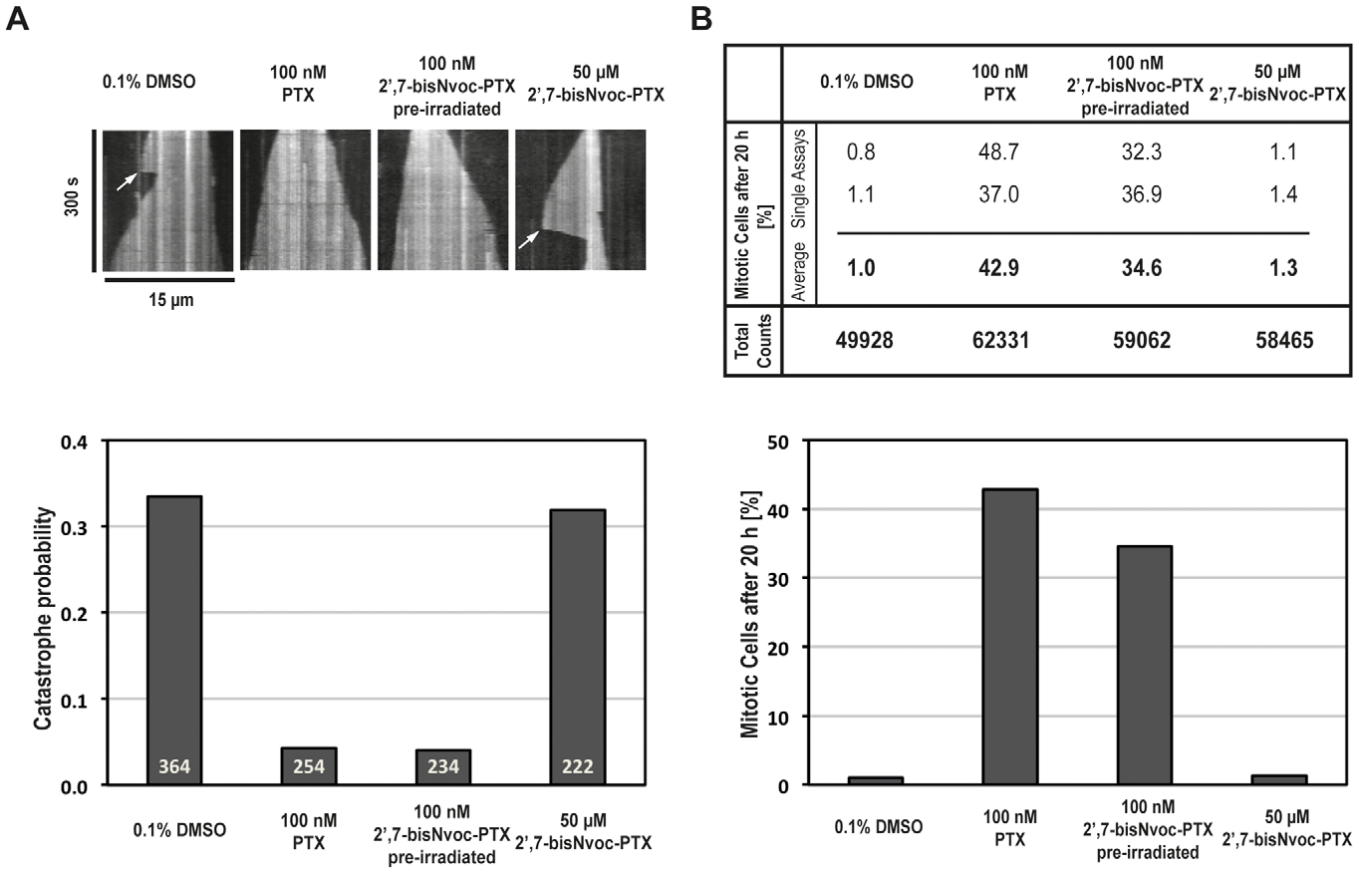
Effect of caged and uncaged 2′,7-bisNvoc-PTX on microtubule catastrophes *in vitro* and on the mitotic index of HeLa cells. (**A**) Upper panel: Representative kymographs of individual Cy5-labeled microtubules as observed by TIRF microscopy in the presence of DMSO, PTX and caged derivatives at the indicated concentrations. White arrows indicate catastrophes. Lower panel: Bar graph with catastrophe probabilities (frequency of catastrophes per microtubule within 5 min) at the different conditions. For each condition microtubules from 3 independent experiments were analyzed. (**B**) Percentage of cells in mitosis after 20 h incubation in medium containing DMSO, PTX and caged derivatives at the indicated concentrations, as measured by flow cytometry. Table: Data from two independent flow cytometric analyses, the average percentage and total count of analyzed cells. Bar graph with the average percentage of cells in mitosis for each condition.

First, using TIRF microscopy, we imaged individual dynamic microtubules *in vitro* in the presence of 50 µM 2′,7-bisNvoc-PTX, 100 nM PTX or pre-irradiated 2′,7-bisNvoc-PTX (Fig. 5A). We determined the catastrophe probability by analyzing kymographs (space-time plots) of single microtubules (Fig. 5A top). 100 nM PTX strongly decreased the number of catastrophes observed over 5 min, as expected [39], very similar to 100 nM pre-irradiated 2′,7-bisNvoc-PTX (Fig. 5A bottom). Remarkably, even at 50 µM 2′,7-bisNvoc-PTX, did not change the catastrophe probability of microtubules *in vitro* compared to the DMSO control (Fig. 5A bottom), supporting our earlier conclusion that this new caged compound is efficiently caged.

Secondly, we performed flow cytometric analysis assays to investigate whether 2′,7-bisNvoc-PTX affects the mitotic index of HeLa cells (Figs. 5B and S7). We found that even 50 µM had no measurable effect on the fraction of cells in mitosis (same low mitotic index as in the DMSO control) (Fig. 5B). In contrast, only 100 nM pre-irradiated 2′,7-bisNvoc-PTX strongly increased the mitotic index, comparable to the effect of 100 nM PTX (Fig. 5B) [40].

In conclusion, these experiments demonstrate that caged 2′,7-bisNvoc-PTX has an at least 500-fold reduced effect on microtubule dynamics *in vitro* and on the cell cycle progression of living cells in comparison to PTX.

### In situ photolysis of caged PTX in cell culture

**Figure 6:**
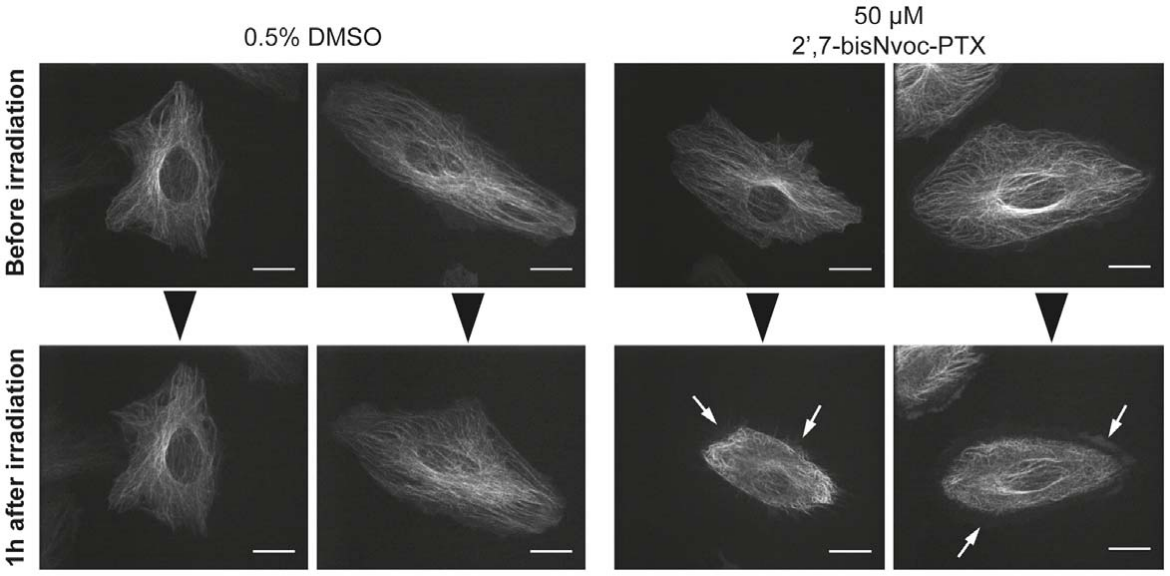
Irradiation of cells in the presence of 2′,7-bisNvoc-PTX under the microscope. Fluorescence microscopy images of HeLa cells stably expressing GFP-tubulin in medium containing DMSO or 2′,7-bisNvoc-PTX. Images show cells before (top) and the same cells 1 h after (bottom) UV irradiation for 5 sec (2 examples for each condition). White arrows indicate the cell edge deprived of microtubules. Scale bar: 20 µm.

In order to test whether in situ uncaging of photo-activatable 2′,7-bisNvoc-PTX in a cell biological assay is possible, we investigated the reorganization of the microtubule cytoskeleton in HeLa cells after irradiation for 5s at 360 nm under the microscope. Cells were imaged before and 1 h after irradiation. In the absence of caged PTX, no aberrant microtubule organization was visible after exposure and cells spread normally. In the presence of 50 µM 2′,7-bisNvoc-PTX, the cytoskeleton of cells was severely disorganized after illumination (Fig. 6), displaying a contracted appearance reminiscent of PTX treated cells. These results demonstrate that double caged PTX can be uncaged in situ and can alter the microtubule assembly in living cells.

### Conclusions

Double caging PTX at positions 2′ and 7 is necessary in order to fully inhibit its activity and to obtain a phototriggerable PTX that can be effective after uncaging at useful concentration ranges. At concentrations up to 50 µM this caged PTX did not affect the microtubule cytoskeleton organization of cultured HeLa cells over 24 h. Short light exposure under a fluorescence microscope was sufficient to trigger the uncaging reaction and allowed controlled delivery of free PTX during imaging. The caged PTX promises to be a new useful molecular tool for cell biologists investigating microtubule dynamics-dependent processes [5], but could also be interesting for photodynamic therapy in medical treatments, where the drug will only be locally liberated at “illuminated” tissues [41].

## Supporting Information

Scheme S1 **Structure of PTX and the caged derivatives.**
(TIF)

Scheme S2 **Synthetic route of the caged PTXs.**
(TIF)

Movie S1 **Uncaging of caged PTXs under the microscope and polymerisation of microtubules** ***in vitro*** **.** Fluorescence microscopy time lapse movies of solutions containing 10 µM Cy5-labeled tubulin without (0.1% DMSO) or with 10 µM caged PTXs as indicated. The 15 min time lapse movies start when the temperature is increased to 35°C. Samples were irradiated with a mercury lamp through the DAPI filter set for 5 sec after the first picture of the movie was aquired. Scale bar: 20 µm.
(AVI)

Movie S2 **Uncaging of 50 µM 2′,7-bisNvoc-PTX under the microscope and polymerisation of microtubules** ***in vitro*** **.** Fluorescence microscopy time lapse movies of solutions containing 10 µM Cy5-labeled tubulin without (0.5% DMSO) or with 50 µM 2′7-bisNvoc-PTX. The 15 min time lapse movies start when the temperature is increased to 35°C, samples were irradiated with a mercury lamp through the DAPI filter set for 5 sec after the first picture of the movie was aquired. Scale bar: 20 µm.
(AVI)

Figure S1 **Stability test.** Analytical HPLC diagrams (left: absorbance at 210 nm, right: absorbance at 380 nm) of 250 µl aliquots of a 100 µM solution of 2′,7-bisNvoc-PTX in 80 mM PIPES (pH 6.9) containing 10% DMSO after storage for 1, 5 and 14 days at r.t. HPLC conditions: 22 min gradient from 50% acetonitrile+5% water+0.1% TFA to 100% acetonitril+5% water+0.1% TFA.
(PDF)

Figure S2 **Irradiation experiments** (**A**) UV-VIS spectra of a 25 µM solution of 2′-Nvoc-PTX upon irradiation with increasing doses. (**B**) Analytical HPLC runs (λ_obs_ 210 nm) of 25 µM solution of PTX (black line), 2′-Nvoc-PTX (red line) and 2′-Nvoc-PTX irradiated 78 min (blue line).
(PDF)

Figure S3 **Irradiation experiments** (**A**) UV-VIS spectra of a solution of 25 µM 7-Nvoc-PTX upon irradiation with increasing doses. (**B**) Analytical HPLC runs (λ_obs_ 210 nm) of 25 µM solution of PTX (black line), 7-Nvoc-PTX (blue line) and 7-Nvoc-PTX irradiated 68 min (red line).
(PDF)

Figure S4 **Irradiation experiments** (**A**) UV-VIS spectra of a solution of 25 µM 2′,7-bisNvoc-PTX upon irradiation with increasing doses. (**B**) Analytical HPLC runs (λ_obs_ 210 nm) of 25 µM solution of PTX (black line), 2′,7-bisNvoc-PTX (red line) and 2′,7-bisNvoc-PTX irradiated 128 min (blue line).
(PDF)

Figure S5 ***In vitro*** **polymerisation of microtubules in the presence of pre-irradiated caged PTXs**. (**A**) Fluorescence microscopy images of a solution containing 10 µM Cy5-labeled tubulin and 10 µM PTX or pre-irradiated caged PTXs (see Methods) or 0.1% DMSO. Images were taken 5 min after temperature increase to 35°C. Scale bars: 20 µm. (**B**) **Quantification of microtubule polymerisation.** Standard deviation of pixel intensities (measure of contrast) as a function of time after the start of polymerization. In contrast to the negative control (0.1% DMSO) all pre-irradiated and uncaged PTXs stimulate microtubule polymerisation.
(PDF)

Figure S6 **Effect of pre-irradiated PTXs on the microtubule cytoskeleton of living cells.** Fluorescence microscopy images of HeLa cells stably expressing GFP-tubulin after 1 h incubation in medium containing 0.1% DMSO, 10 µM PTX or pre-irradiated and uncaged PTX derivatives. White arrows indicate the cell edge deprived of microtubules. Scale bars: 20 µm.
(PDF)

Figure S7 **Dot plots of the FACS analysis data of HeLa cells.** Data are shown from two independent experiments of HeLa cells after 20 h incubation in medium containing DMSO, PTX and caged derivatives at indicated concentrations respectively. The plots show the anti-phospho histone H3 serine 10 labeling intensities (indicative of the mitotic cells) versus the intensity of the propidium iodide nucleic acid stain (indicative of the total amount of DNA per cell).
(PDF)

Figure S8 **NMR spectra of caged PTXs intermediates and products.**
(PDF)

Table S1 **Detection of photolytic products.** Molar composition of a solution of 2′,7-bisNvoc-PTX (1 mM, 5% water in acetonitrile) after different irradiation times, as determined by analysis of HPLC graphs (Figure 1B in manuscript). The retention times (r.t.) for the different variants were: 2′-Nvoc-PTX (r.t. = 16.7 min), 7-Nvoc-PTX (r.t. = 18.2 min), 2′,7-bisNvoc-PTX (r.t. = 21.3 min), PTX (r.t. = 11.1 min).
(PDF)
