# Supplementary figures and images for: Phototriggerable 2′,7-Caged Paclitaxel
Source: PLoS One. 2012 Sep 6;7(9):e43657. doi: 10.1371/journal.pone.0043657 (PMC3435387; doi:10.1371/journal.pone.0043657)

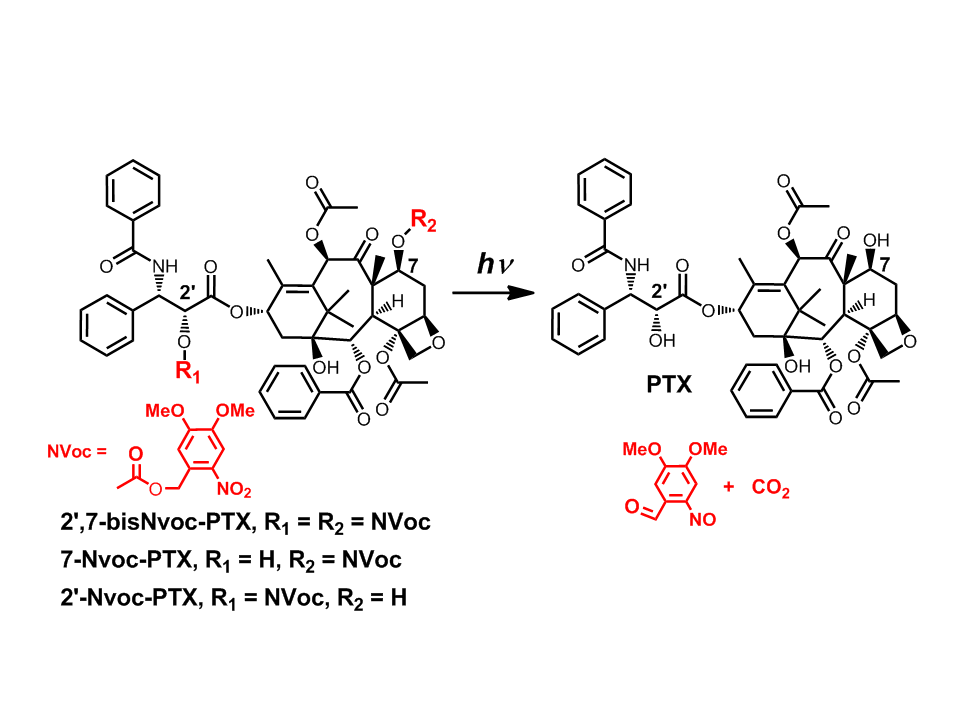

Supplement: Scheme S1 — Structure of PTX and the caged derivatives. (TIF) [file pone.0043657.s001.tif]
