# Supplementary material for: Phototriggerable 2′,7-Caged Paclitaxel
Source: PLoS One. 2012 Sep 6;7(9):e43657. doi: 10.1371/journal.pone.0043657 (PMC3435387; doi:10.1371/journal.pone.0043657)
Supplement: Figure S1 — Stability test. Analytical HPLC diagrams (left: absorbance at 210 nm, right: absorbance at 380 nm) of 250 µl aliquots of a 100 µM solution of 2′,7-bisNvoc-PTX in 80 mM PIPES (pH 6.9) containing 10% DMSO after storage for 1, 5 and 14 days at r.t. HPLC conditions: 22 min gradient from 50% acetonitrile+5% water+0.1% TFA to 100% acetonitril+5% water+0.1% TFA. (PDF) [file pone.0043657.s005.pdf]

SUPPORTING INFORMATION

Phototriggerable 2',7-caged Paclitaxel

Radu A. Gropeanu[1], Hella Baumann[2], Sandra Ritz[1], Volker Mailänder[1,3], Thomas Surrey[2], Aránzazu del Campo[1]*

[1] Max-Planck-Institut für Polymerforschung, Ackermannweg 10, 55128 Mainz, Germany. Tel +49 6131 379563; Fax +49 6131 379271, e-mail: delcampo@mpip-mainz.mpg.de

[2] Microtubule Cytoskeleton Laboratory, London Research Institute, Lincoln's Inn Fields Laboratories, 44 Lincoln's Inn Fields, London WC2A 3LY, United Kingdom

[3] 3rd Department of Medicine (Hematology, Oncology, and Pneumology), University Medical Center of Johannes Gutenberg-University Mainz, Langenbeckstr. 1, 55131 Mainz, Germany

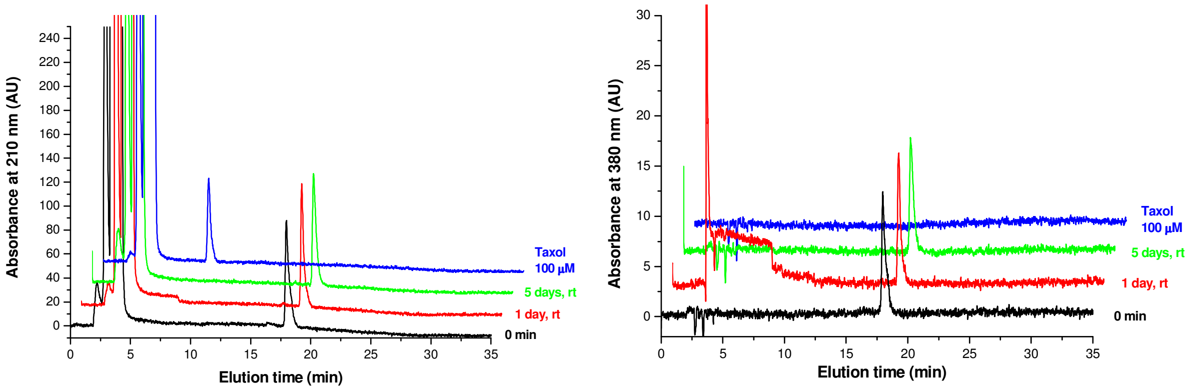

Figure S1. Stability test. Analytical HPLC diagrams (left: absorbance at 210 nm, right: absorbance at 380 nm) of 250 µl aliquots of a 100 µM solution of 2',7-bisNvoc-PTX in 80 mM PIPES (pH 6.9) containing 10% DMSO after storage for 1, 5 and 14 days at r.t. HPLC conditions: 22 min gradient from 50% acetonitrile +5 % water + 0.1% TFA to 100% acetonitril +5 % water + 0.1% TFA.
