# Supplementary material for: Phototriggerable 2′,7-Caged Paclitaxel
Source: PLoS One. 2012 Sep 6;7(9):e43657. doi: 10.1371/journal.pone.0043657 (PMC3435387; doi:10.1371/journal.pone.0043657)
Supplement: Figure S2 — Irradiation experiments (A) UV-VIS spectra of a 25 µM solution of 2′-Nvoc-PTX upon irradiation with increasing doses. (B) Analytical HPLC runs (λobs 210 nm) of 25 µM solution of PTX (black line), 2′-Nvoc-PTX (red line) and 2′-Nvoc-PTX irradiated 78 min (blue line). (PDF) [file pone.0043657.s006.pdf]

SUPPORTING INFORMATION

Phototriggerable 2',7-caged Paclitaxel

Radu A. Gropeanu[1], Hella Baumann[2], Sandra Ritz[1], Volker Mailänder[1,3], Thomas Surrey[2], Aránzazu del Campo[1]*

[1] Max-Planck-Institut für Polymerforschung, Ackermannweg 10, 55128 Mainz, Germany. Tel +49 6131 379563; Fax +49 6131 379271, e-mail: delcampo@mpip-mainz.mpg.de

[2] Microtubule Cytoskeleton Laboratory, London Research Institute, Lincoln's Inn Fields Laboratories, 44 Lincoln's Inn Fields, London WC2A 3LY, United Kingdom

[3] 3rd Department of Medicine (Hematology, Oncology, and Pneumology), University Medical Center of Johannes Gutenberg-University Mainz, Langenbeckstr. 1, 55131 Mainz, Germany

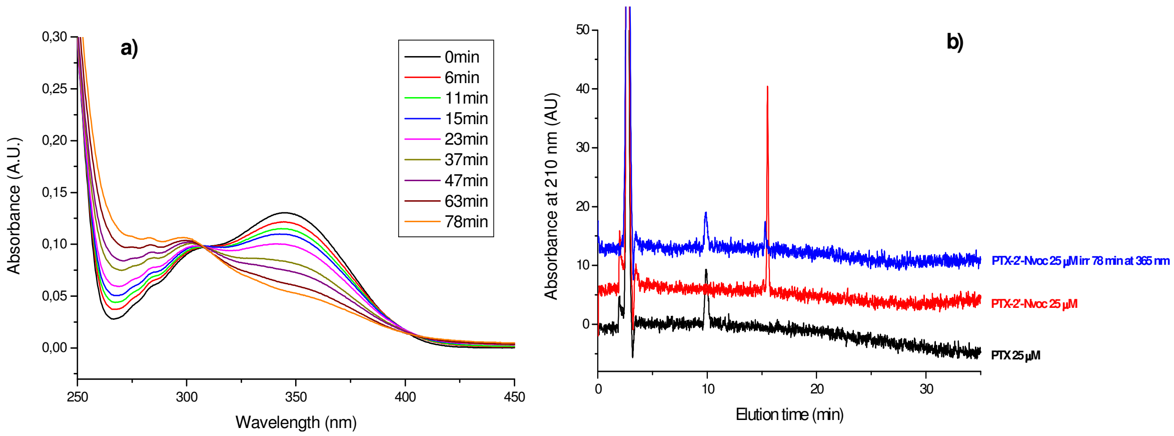

Figure S2. **Irradiation experiments (A)** UV-VIS spectra of a 25 µM solution of 2'-Nvoc-PTX upon irradiation with increasing doses. **(B)** Analytical HPLC runs (λ_{obs} 210 nm) of 25 µM solution of PTX (black line), 2'-Nvoc-PTX (red line) and 2'-Nvoc-PTX irradiated 78 min (blue line).
