# Supplementary material for: Phototriggerable 2′,7-Caged Paclitaxel
Source: PLoS One. 2012 Sep 6;7(9):e43657. doi: 10.1371/journal.pone.0043657 (PMC3435387; doi:10.1371/journal.pone.0043657)
Supplement: Figure S5 — In vitro polymerisation of microtubules in the presence of pre-irradiated caged PTXs. (A) Fluorescence microscopy images of a solution containing 10 µM Cy5-labeled tubulin and 10 µM PTX or pre-irradiated caged PTXs (see Methods) or 0.1% DMSO. Images were taken 5 min after temperature increase to 35°C. Scale bars: 20 µm. (B) Quantification of microtubule polymerisation. Standard deviation of pixel intensities (measure of contrast) as a function of time after the start of polymerization. In contrast to the negative control (0.1% DMSO) all pre-irradiated and uncaged PTXs stimulate microtubule polymerisation. (PDF) [file pone.0043657.s009.pdf]

SUPPORTING INFORMATION

Phototriggerable 2’,7-caged Paclitaxel

Radu A. Gropeanu[1], Hella Baumann[2], Sandra Ritz[1], Volker Mailänder[1,3], Thomas Surrey[2], Aránzazu del Campo[1]*

[1] Max-Planck-Institut für Polymerforschung, Ackermannweg 10, 55128 Mainz, Germany. Tel +49 6131 379563; Fax +49 6131 379271, e-mail: delcampo@mpip-mainz.mpg.de

[2] Microtubule Cytoskeleton Laboratory, London Research Institute, Lincoln's Inn Fields Laboratories, 44 Lincoln's Inn Fields, London WC2A 3LY, United Kingdom

[3] 3rd Department of Medicine (Hematology, Oncology, and Pneumology), University Medical Center of Johannes Gutenberg-University Mainz, Langenbeckstr. 1, 55131 Mainz, Germany

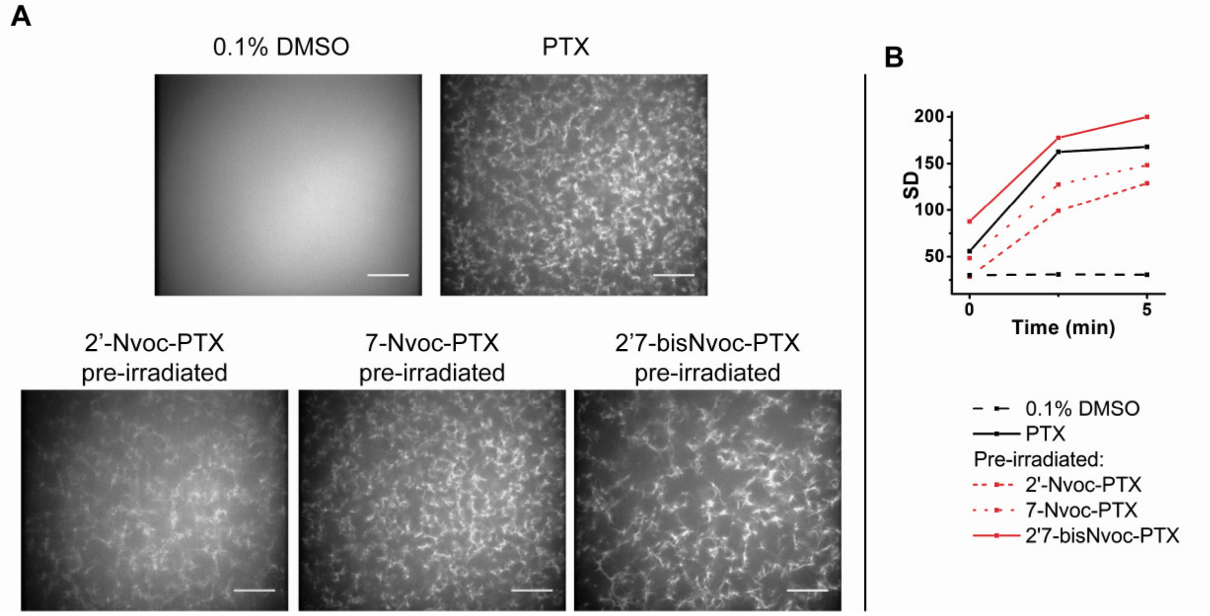

Figure S5. ***In vitro* polymerisation of microtubules in the presence of pre-irradiated caged PTXs. (A)** Fluorescence microscopy images of a solution containing 10 µM Cy5-labeled tubulin and 10 µM PTX or pre-irradiated caged PTXs (see Methods) or 0.1% DMSO. Images were taken 5 min after temperature increase to 35 °C. Scale bars: 20 µm. **(B) Quantification of microtubule polymerisation.** Standard deviation of pixel intensities (measure of contrast) as a function of time after the start of polymerization. In contrast to the negative control (0.1% DMSO) all pre-irradiated and uncaged PTXs stimulate microtubule polymerisation.
