# Supplementary material for: Phototriggerable 2′,7-Caged Paclitaxel
Source: PLoS One. 2012 Sep 6;7(9):e43657. doi: 10.1371/journal.pone.0043657 (PMC3435387; doi:10.1371/journal.pone.0043657)
Supplement: Figure S6 — Effect of pre-irradiated PTXs on the microtubule cytoskeleton of living cells. Fluorescence microscopy images of HeLa cells stably expressing GFP-tubulin after 1 h incubation in medium containing 0.1% DMSO, 10 µM PTX or pre-irradiated and uncaged PTX derivatives. White arrows indicate the cell edge deprived of microtubules. Scale bars: 20 µm. (PDF) [file pone.0043657.s010.pdf]

## SUPPORTING INFORMATION

# Phototriggerable 2',7-caged Paclitaxel

**Radu A. Gropeanu[1], Hella Baumann[2], Sandra Ritz[1], Volker Mailänder[1,3], Thomas Surrey[2], Aránzazu del Campo[1]***

[1] Max-Planck-Institut für Polymerforschung, Ackermannweg 10, 55128 Mainz, Germany. Tel +49 6131 379563; Fax +49 6131 379271, e-mail: delcampo@mpip-mainz.mpg.de

[2] Microtubule Cytoskeleton Laboratory, London Research Institute, Lincoln's Inn Fields Laboratories, 44 Lincoln's Inn Fields, London WC2A 3LY, United Kingdom

[3] 3rd Department of Medicine (Hematology, Oncology, and Pneumology), University Medical Center of Johannes Gutenberg-University Mainz, Langenbeckstr. 1, 55131 Mainz, Germany

### Effect of caged and uncaged PTX on living cells

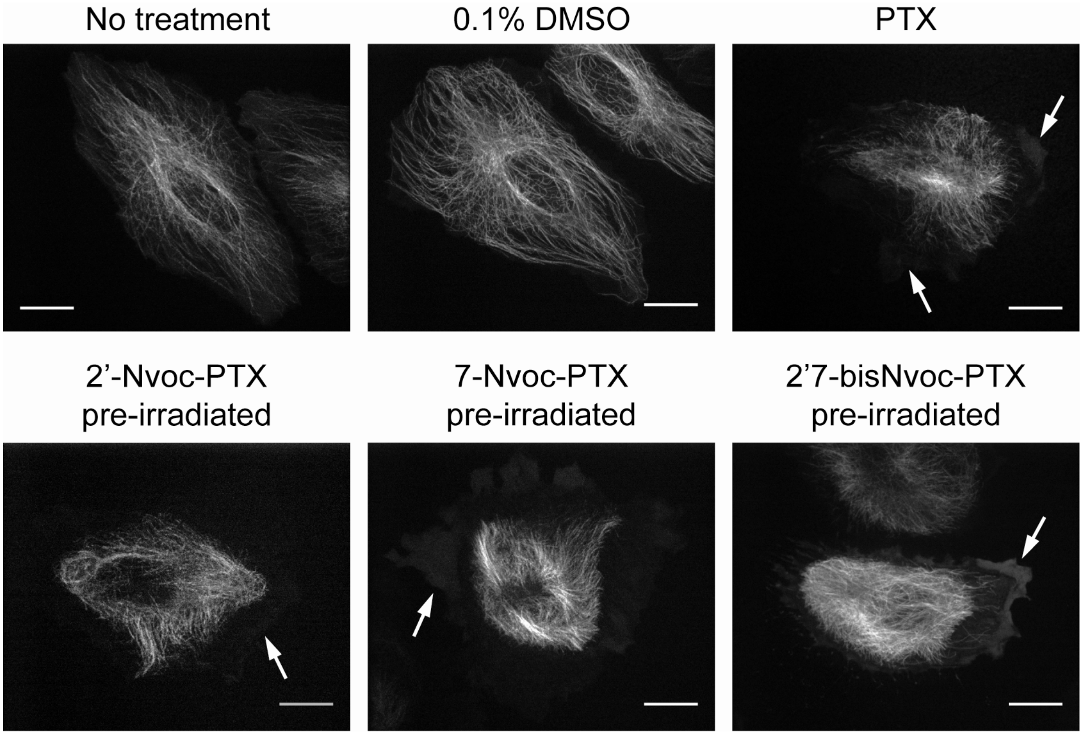


**Figure S6. Effect of pre-irradiated PTXs on the microtubule cytoskeleton of living cells.** Fluorescence microscopy images of HeLa cells stably expressing GFP-tubulin after 1 h incubation in medium containing 0.1% DMSO, 10µM PTX or pre-irradiated and uncaged PTX derivatives. White arrows indicate the cell edge deprived of microtubules. Scale bars: 20 µm.
