# Supplementary material for: Phototriggerable 2′,7-Caged Paclitaxel
Source: PLoS One. 2012 Sep 6;7(9):e43657. doi: 10.1371/journal.pone.0043657 (PMC3435387; doi:10.1371/journal.pone.0043657)
Supplement: Figure S7 — Dot plots of the FACS analysis data of HeLa cells. Data are shown from two independent experiments of HeLa cells after 20 h incubation in medium containing DMSO, PTX and caged derivatives at indicated concentrations respectively. The plots show the anti-phospho histone H3 serine 10 labeling intensities (indicative of the mitotic cells) versus the intensity of the propidium iodide nucleic acid stain (indicative of the total amount of DNA per cell). (PDF) [file pone.0043657.s011.pdf]

SUPPORTING INFORMATION

Phototriggerable 2',7-caged Paclitaxel

Radu A. Gropeanu[1], Hella Baumann[2], Sandra Ritz[1], Volker Mailänder[1,3], Thomas Surrey[2], Aránzazu del Campo[1]*

[1] Max-Planck-Institut für Polymerforschung, Ackermannweg 10, 55128 Mainz, Germany. Tel +49 6131 379563; Fax +49 6131 379271, e-mail: delcampo@mpip-mainz.mpg.de

[2] Microtubule Cytoskeleton Laboratory, London Research Institute, Lincoln's Inn Fields Laboratories, 44 Lincoln's Inn Fields, London WC2A 3LY, United Kingdom

[3] 3rd Department of Medicine (Hematology, Oncology, and Pneumology), University Medical Center of Johannes Gutenberg-University Mainz, Langenbeckstr. 1, 55131 Mainz, Germany

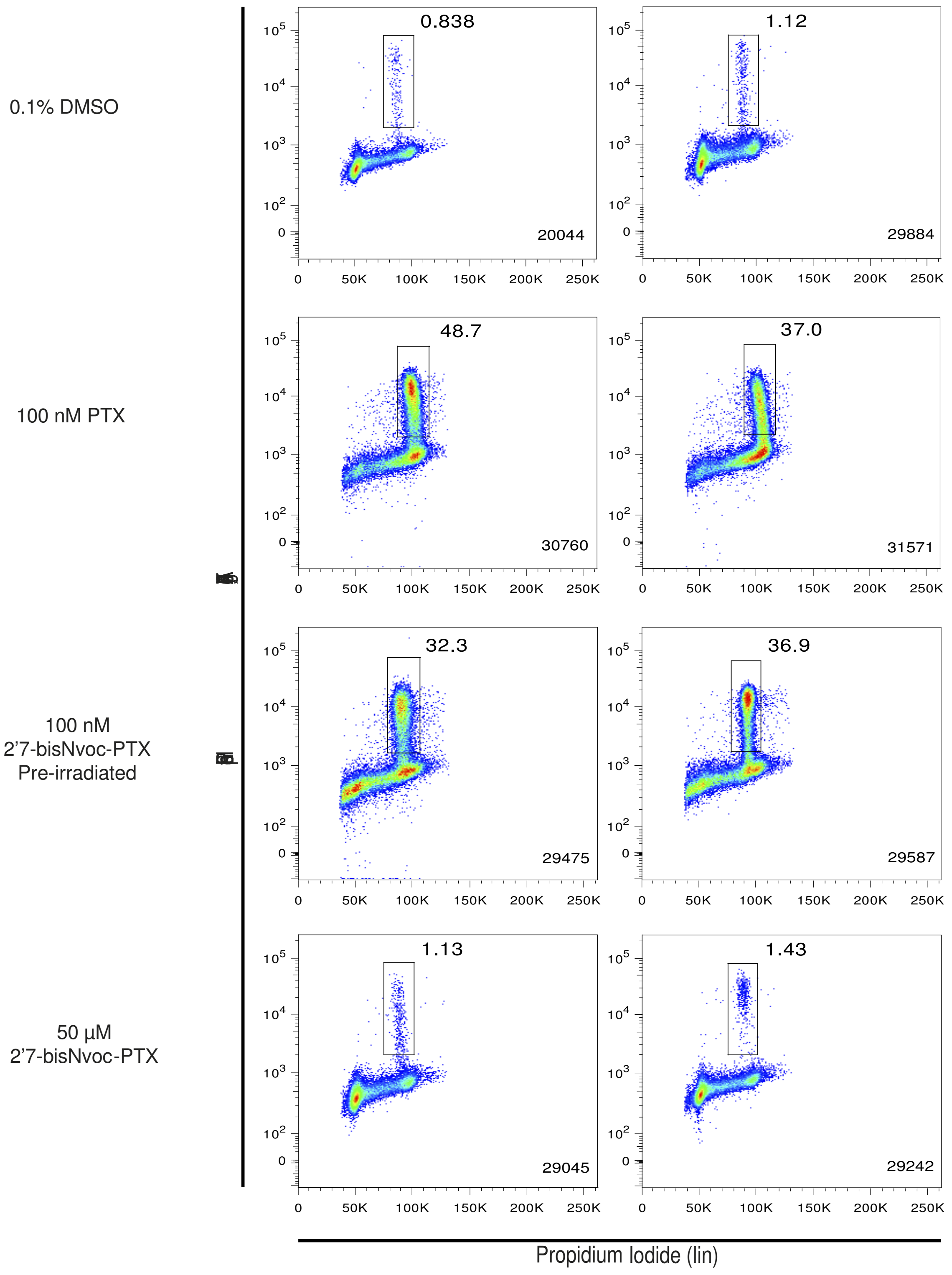

Figure S7. Dot plots of the FACS analysis data of HeLa cells. Data are shown from two independent experiments of HeLa cells after 20 h incubation in medium containing DMSO, PTX and caged derivatives at indicated concentrations respectively. The plots show the anti-phospho histone H3 serine 10 labeling intensities (indicative of the mitotic cells) versus the intensity of the propidium iodide nucleic acid stain (indicative of the total amount of DNA per cell).
