# Supplementary material for: Phototriggerable 2′,7-Caged Paclitaxel
Source: PLoS One. 2012 Sep 6;7(9):e43657. doi: 10.1371/journal.pone.0043657 (PMC3435387; doi:10.1371/journal.pone.0043657)
Supplement: Figure S8 — NMR spectra of caged PTXs intermediates and products. (PDF) [file pone.0043657.s012.pdf]

SUPPORTING INFORMATION

Phototriggerable 2',7-caged Paclitaxel

Radu A. Gropeanu[1], Hella Baumann[2], Sandra Ritz[1], Volker Mailänder[1,3], Thomas Surrey[2], Aránzazu del Campo[1]*

[1] Max-Planck-Institut für Polymerforschung, Ackermannweg 10, 55128 Mainz, Germany. Tel +49 6131 379563; Fax +49 6131 379271, e-mail: delcampo@mpip-mainz.mpg.de

[2] Microtubule Cytoskeleton Laboratory, London Research Institute, Lincoln's Inn Fields Laboratories, 44 Lincoln's Inn Fields, London WC2A 3LY, United Kingdom

[3] 3rd Department of Medicine (Hematology, Oncology, and Pneumology), University Medical Center of Johannes Gutenberg-University Mainz, Langenbeckstr. 1, 55131 Mainz, Germany

Figure S8: NMR Spectra of caged PTXs and intermediates

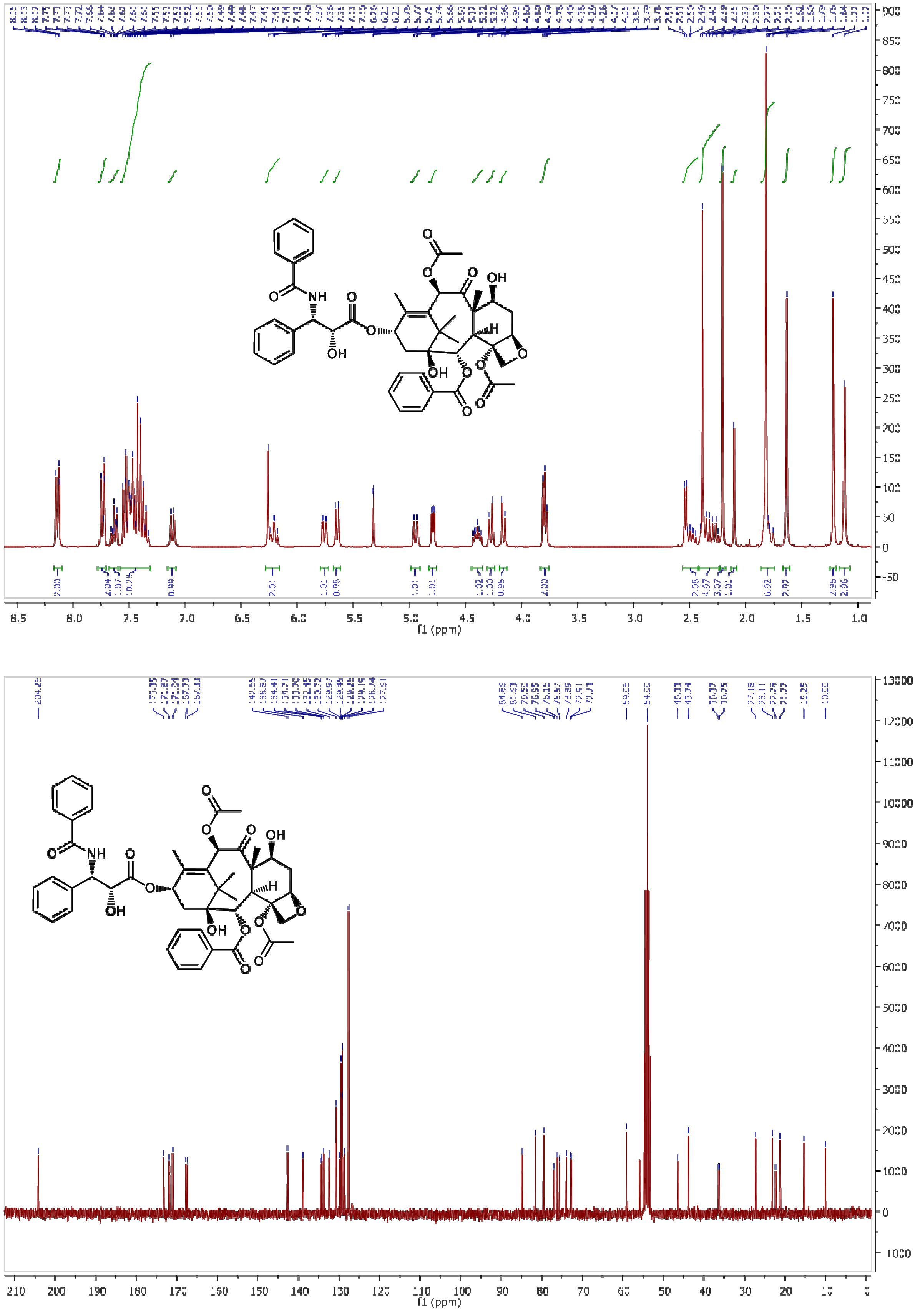

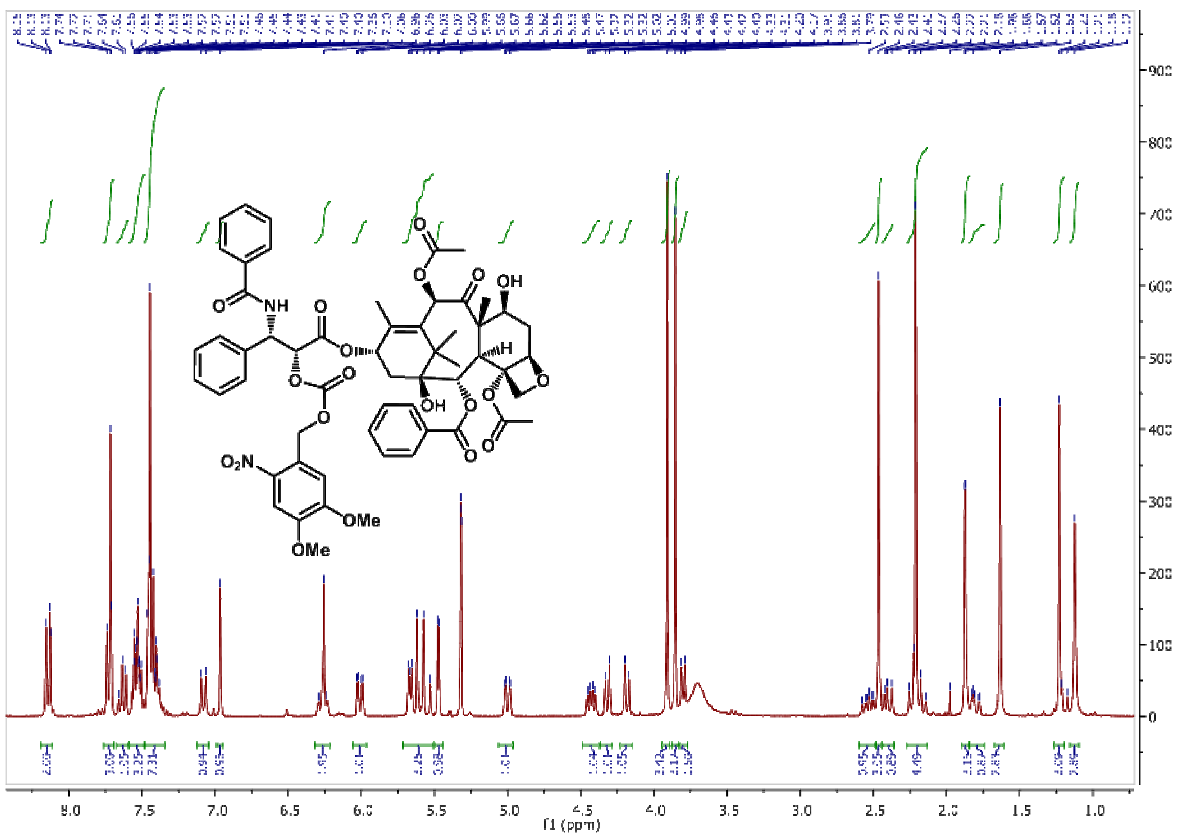
f1 (ppm)

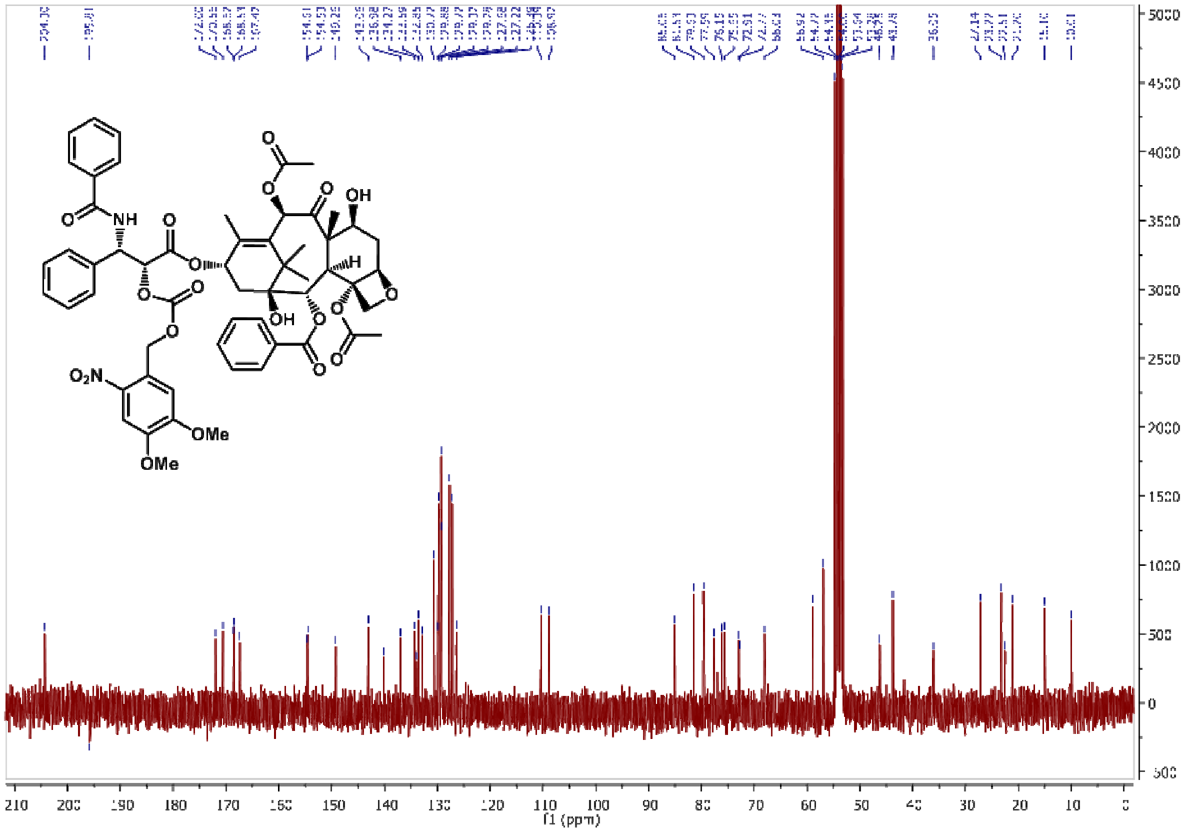
f1 (ppm)

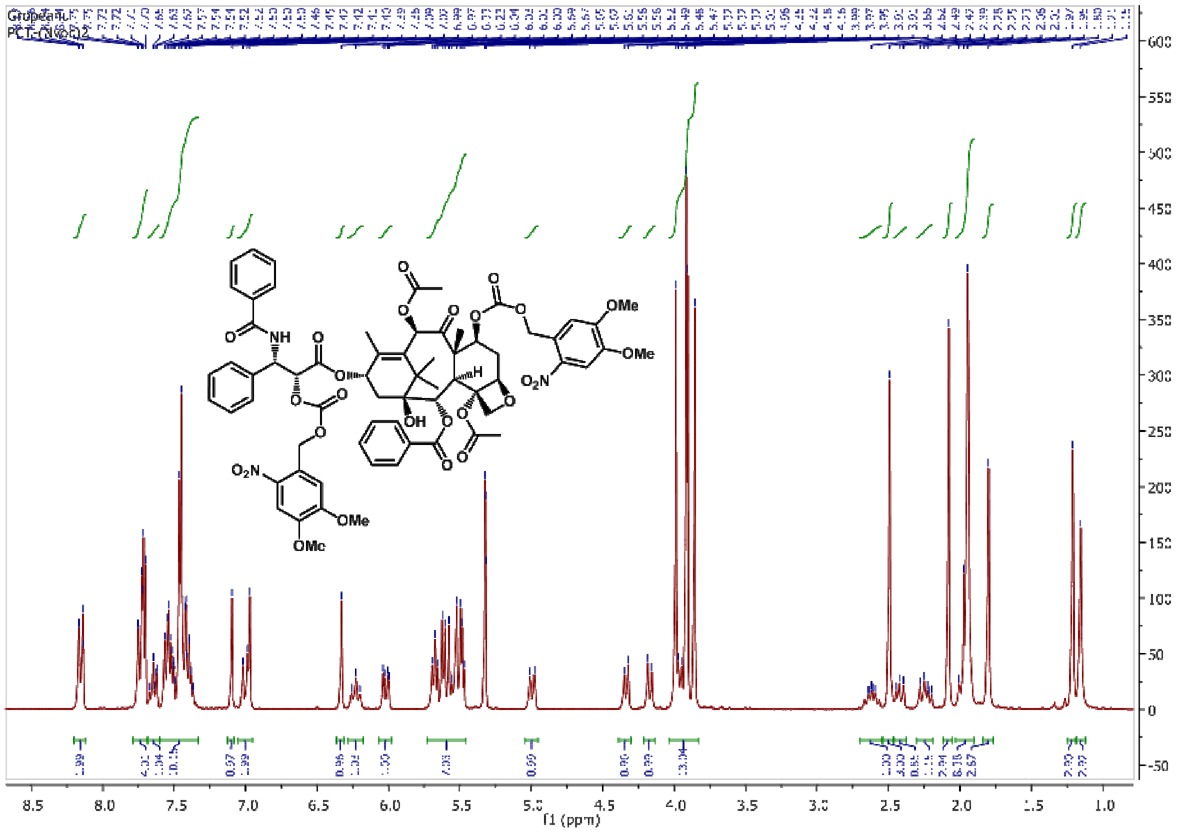
NH
OH
OMe
OMe
OMe
OMe
f1 (ppm)

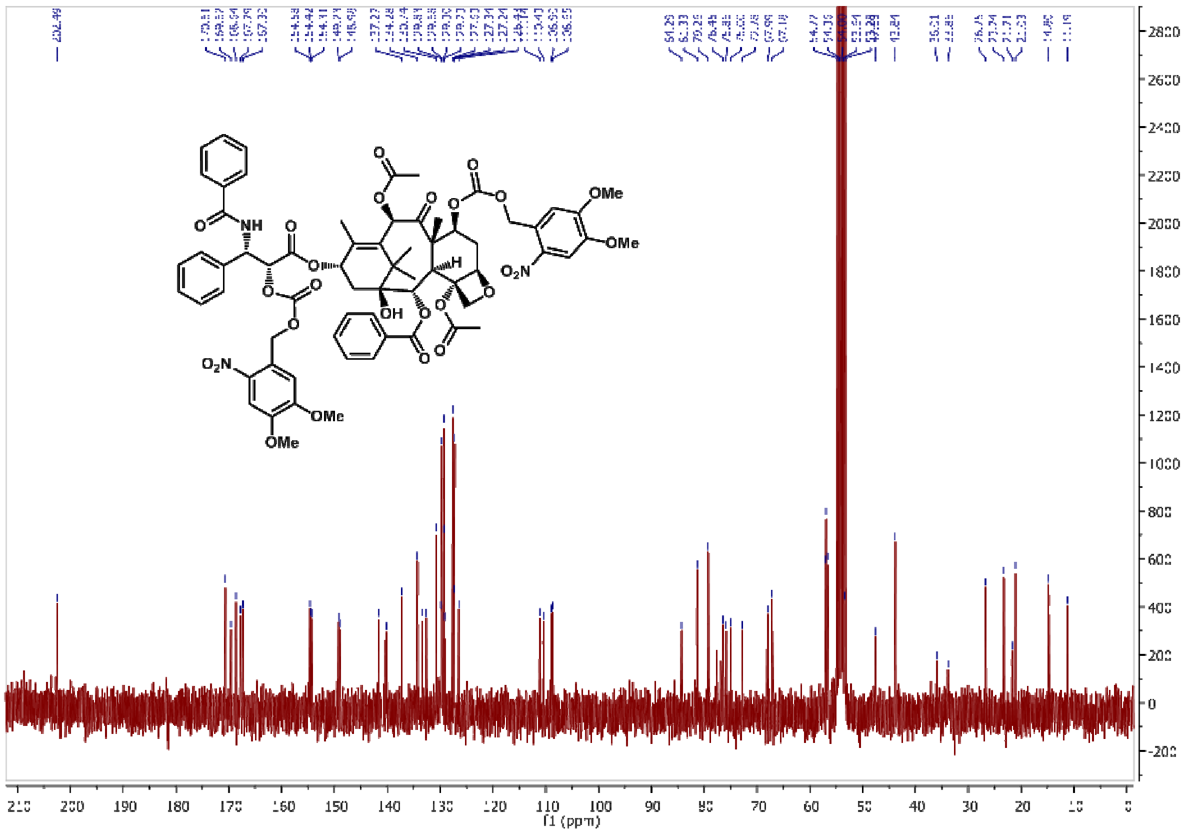
NH
OH
OMe
OMe
OMe
OMe
f1 (ppm)

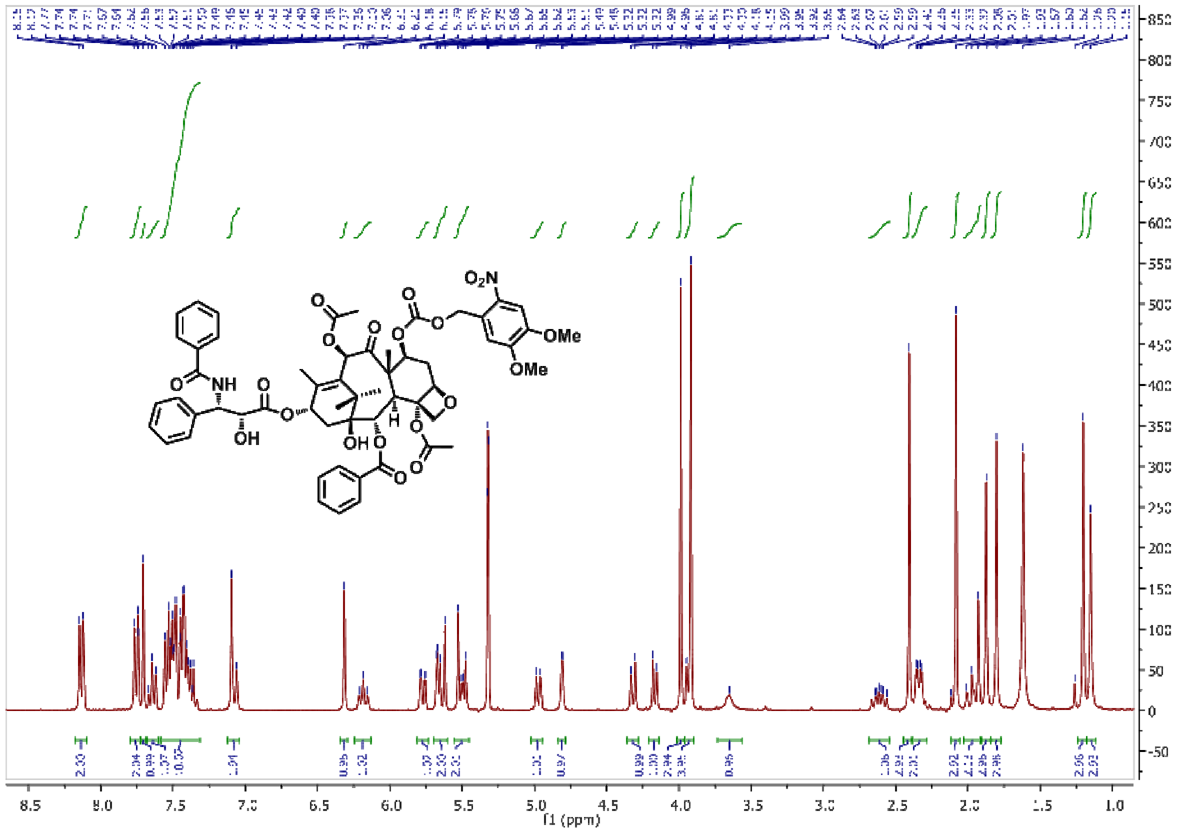

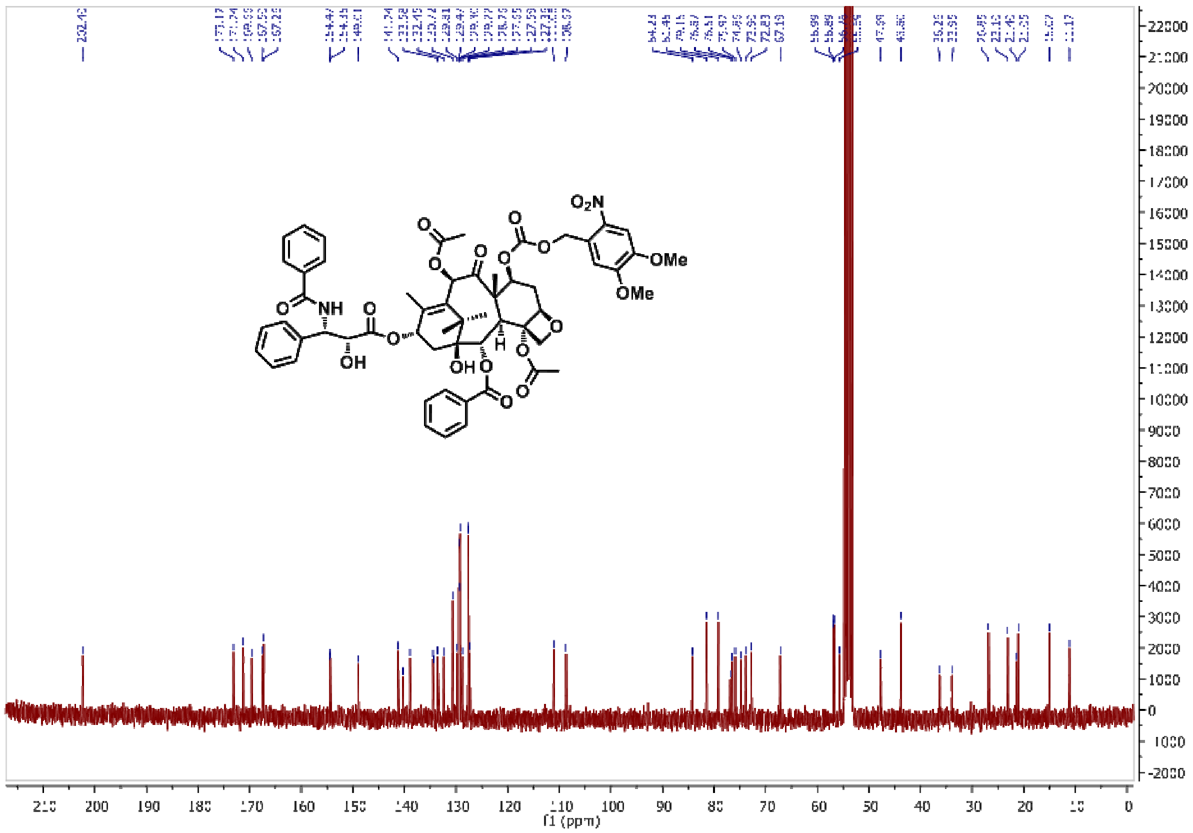

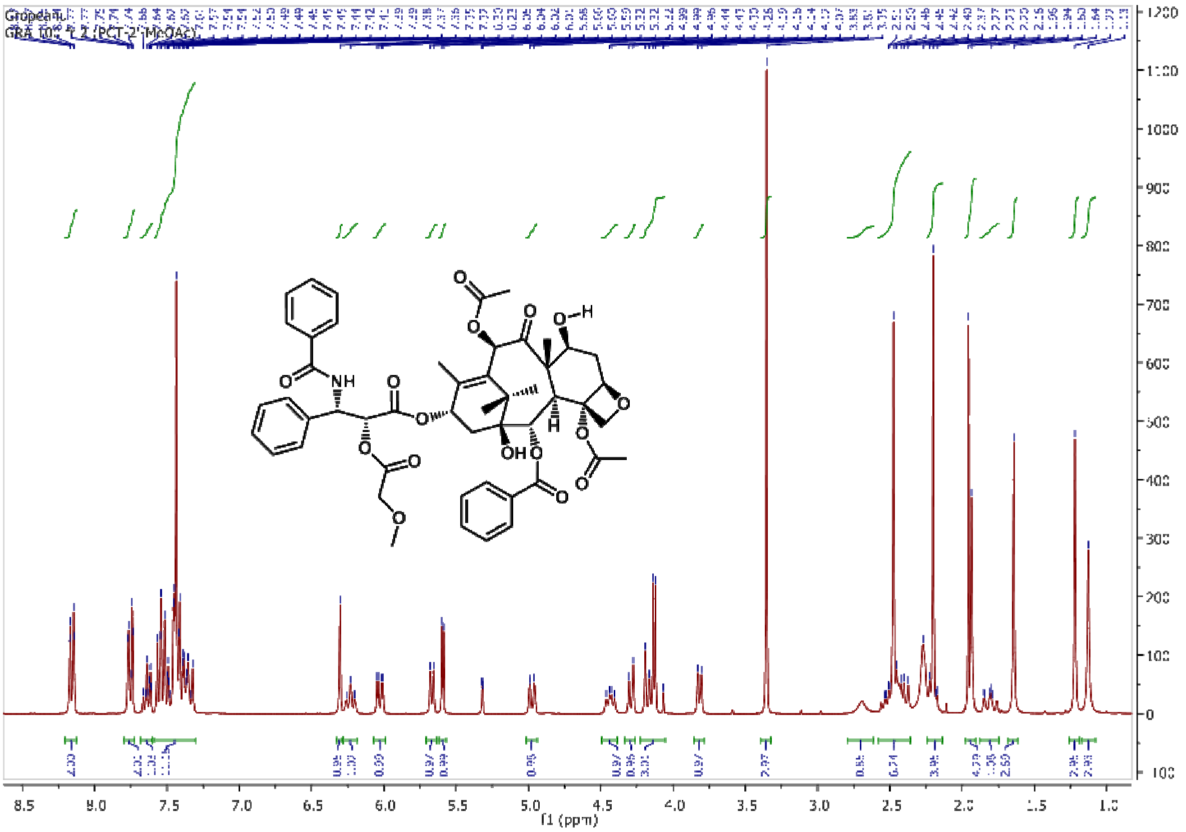
f1 (ppm)

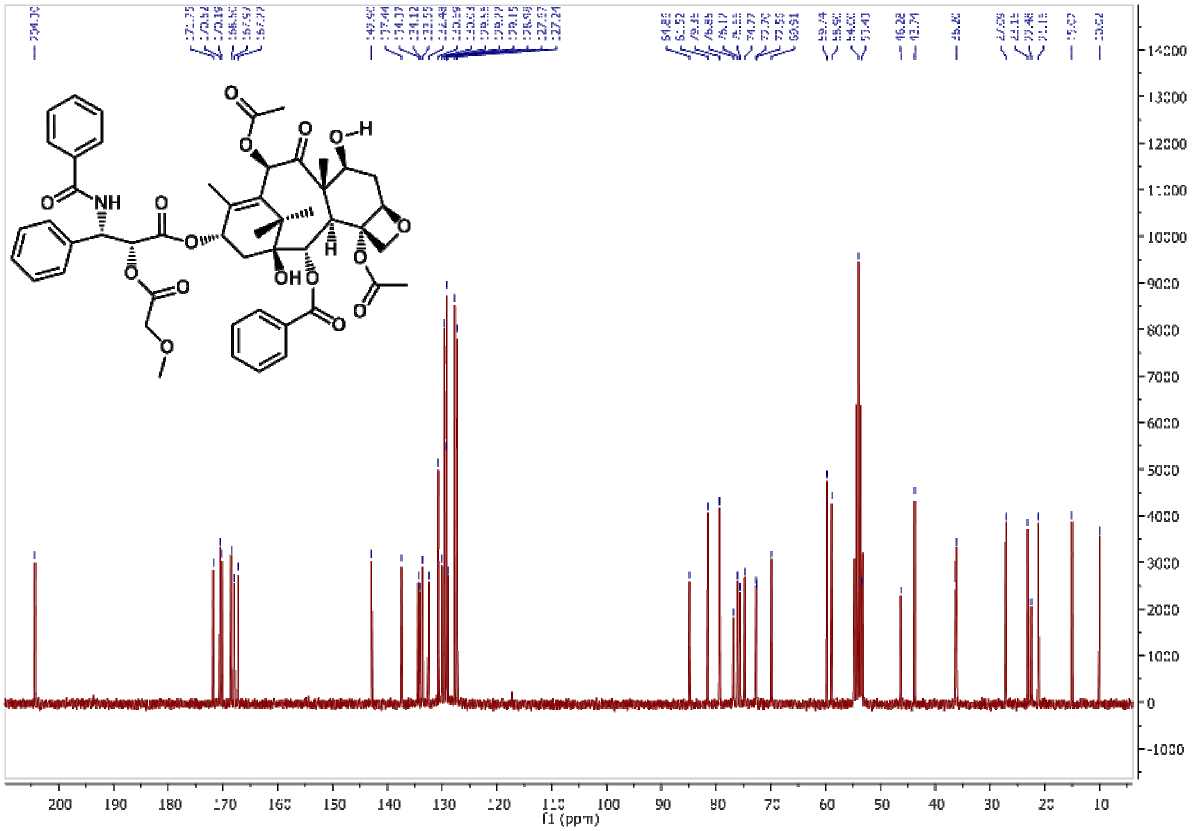
f1 (ppm)

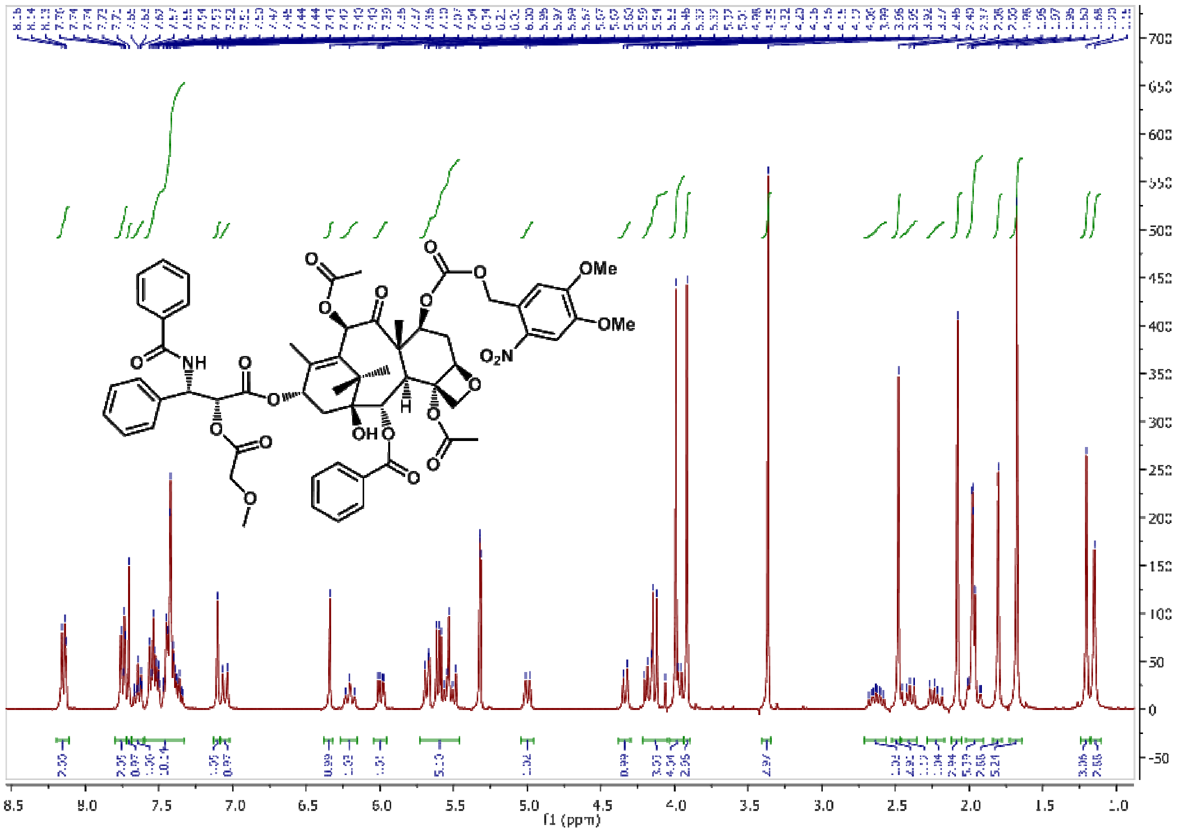
OMe
OMe
O2N
NH
OH
H
f1 (ppm)

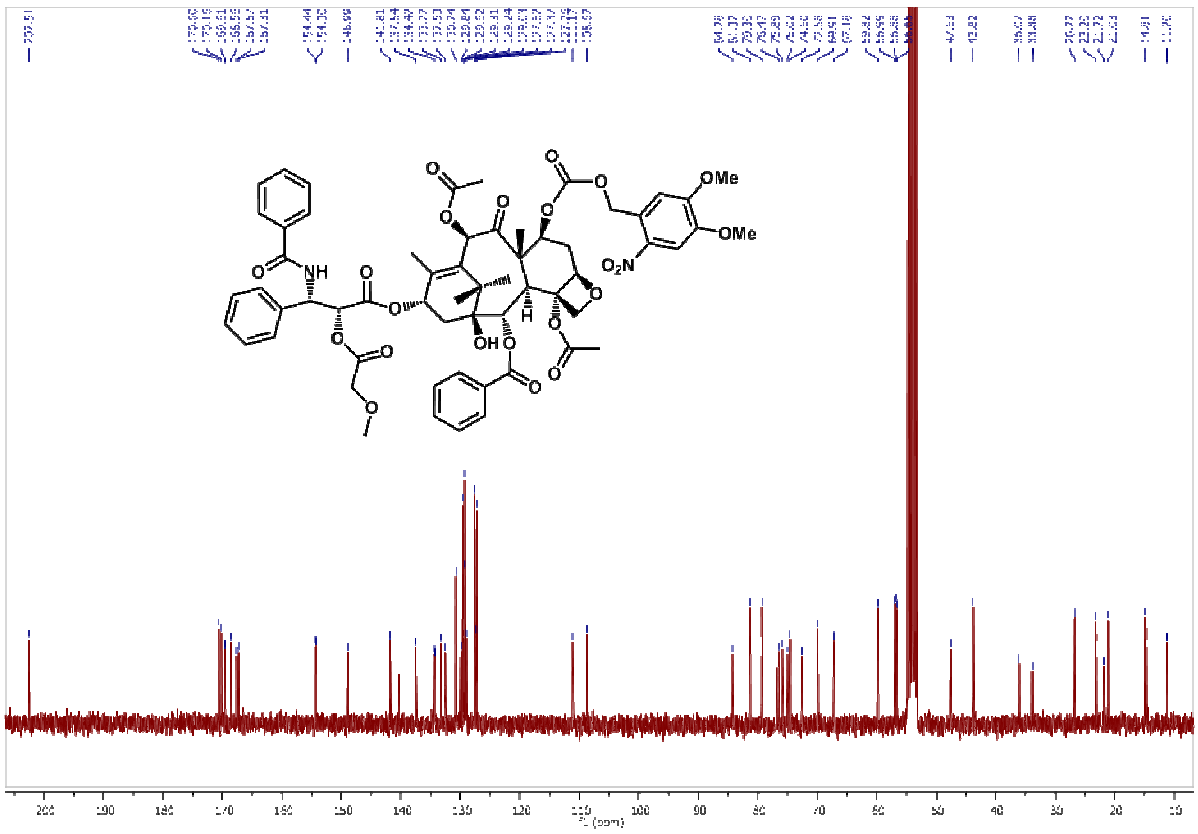
OMe
OMe
O2N
NH
OH
H
