# Supplementary material for: Phototriggerable 2′,7-Caged Paclitaxel
Source: PLoS One. 2012 Sep 6;7(9):e43657. doi: 10.1371/journal.pone.0043657 (PMC3435387; doi:10.1371/journal.pone.0043657)
Supplement: Table S1 — Detection of photolytic products. Molar composition of a solution of 2′,7-bisNvoc-PTX (1 mM, 5% water in acetonitrile) after different irradiation times, as determined by analysis of HPLC graphs (Figure 1B in manuscript). The retention times (r.t.) for the different variants were: 2′-Nvoc-PTX (r.t. = 16.7 min), 7-Nvoc-PTX (r.t. = 18.2 min), 2′,7-bisNvoc-PTX (r.t. = 21.3 min), PTX (r.t. = 11.1 min). (PDF) [file pone.0043657.s013.pdf]

SUPPORTING INFORMATION

Phototriggerable 2’,7-caged Paclitaxel

Radu A. Gropeanu[1], Hella Baumann[2], Sandra Ritz[1], Volker Mailänder[1,3], Thomas Surrey[2], Aránzazu del Campo[1]*

[1] Max-Planck-Institut für Polymerforschung, Ackermannweg 10, 55128 Mainz, Germany. Tel +49 6131 379563; Fax +49 6131 379271, e-mail: delcampo@mpip-mainz.mpg.de

[2] Microtubule Cytoskeleton Laboratory, London Research Institute, Lincoln's Inn Fields Laboratories, 44 Lincoln's Inn Fields, London WC2A 3LY, United Kingdom

[3] 3rd Department of Medicine (Hematology, Oncology, and Pneumology), University Medical Center of Johannes Gutenberg-University Mainz, Langenbeckstr. 1, 55131 Mainz, Germany

Table S1. **Detection of photolytic products.** Molar composition of a solution of 2’,7-bisNvoc-PTX (1 mM, 5% water in acetonitrile) after different irradiation times, as determined by analysis of HPLC graphs (Figure 1B in manuscript). The retention times (r.t.) for the different variants were: 2’-Nvoc-PTX (r.t.= 16.7 min), 7-Nvoc-PTX (r.t. = 18.2 min), 2’,7-bisNvoc-PTX (r.t. = 21.3 min), PTX (r.t. = 11.1 min).

Irradiation Time (min)	PTX (%)	2’-Nvoc-PTX (%)	7-Nvoc-PTX (%)	2’,7-bisNvoc-PTX (%)
0	0	0	0	100
60	0	6.3	3.2	90.5
270	1.7	17.4	8.6	72.3
450	6.0	27.6	15.3	51.1
570	10.6	32.3	14.9	42.2
735	18.5	35.6	18.4	27.5
820	21.2	38.1	16.4	24.3
1585	60.5	25.6	7.8	6.1
